# Molecular modeling and phylogenetic analyses highlight the role of amino acid 347 of the N1 subtype neuraminidase in influenza virus host range and interspecies adaptation

**DOI:** 10.3389/fmicb.2023.1309156

**Published:** 2023-12-19

**Authors:** Stefano Elli, Giuseppina Raffaini, Marco Guerrini, Sergei Kosakovsky Pond, Mikhail Matrosovich

**Affiliations:** ^1^Istituto di Ricerche Chimiche e Biochimiche ‘G. Ronzoni’, Milan, Italy; ^2^Department of Chemistry, Materials, and Chemical Engineering “Giulio Natta”, Politecnico di Milano, Milan, Italy; ^3^Institute for Genomics and Evolutionary Medicine, Temple University, Philadelphia, PA, United States; ^4^Institute of Virology, Philipps University, Marburg, Germany

**Keywords:** influenza, neuraminidase, substrate specificity, MD simulation, natural selection, H5N1

## Abstract

The N1 neuraminidases (NAs) of avian and pandemic human influenza viruses contain tyrosine and asparagine, respectively, at position 347 on the rim of the catalytic site; the biological significance of this difference is not clear. Here, we used molecular dynamics simulation to model the effects of amino acid 347 on N1 NA interactions with sialyllacto-N-tetraoses 6’SLN-LC and 3’SLN-LC, which represent NA substrates in humans and birds, respectively. Our analysis predicted that Y347 plays an important role in the NA preference for the avian-type substrates. The Y347N substitution facilitates hydrolysis of human-type substrates by resolving steric conflicts of the Neu5Ac2–6Gal moiety with the bulky side chain of Y347, decreasing the free energy of substrate binding, and increasing the solvation of the Neu5Ac2–6Gal bond. Y347 was conserved in all N1 NA sequences of avian influenza viruses in the GISAID EpiFlu database with two exceptions. First, the Y347F substitution was present in the NA of a specific H6N1 poultry virus lineage and was associated with the substitutions G228S and/or E190V/L in the receptor-binding site (RBS) of the hemagglutinin (HA). Second, the highly pathogenic avian H5N1 viruses of the Gs/Gd lineage contained sporadic variants with the NA substitutions Y347H/D, which were frequently associated with substitutions in the HA RBS. The Y347N substitution occurred following the introductions of avian precursors into humans and pigs with N/D347 conserved during virus circulation in these hosts. Comparative evolutionary analysis of site 347 revealed episodic positive selection across the entire tree and negative selection within most host-specific groups of viruses, suggesting that substitutions at NA position 347 occurred during host switches and remained under pervasive purifying selection thereafter. Our results elucidate the role of amino acid 347 in NA recognition of sialoglycan substrates and emphasize the significance of substitutions at position 347 as a marker of host range and adaptive evolution of influenza viruses.

## 1 Introduction

Wild aquatic birds are a primary natural reservoir of influenza A viruses (IVs). Transmission and occasional adaptation of aquatic bird viruses to other avian and mammalian species, with or without gene reassortment and/or additional host-switching events, led to the formation of the known variety of HxNy host-specific lineages of IVs, such as H5N1, H7N9 and H9N2 poultry IVs, H1N1 “classical” and “avian-like” swine IVs, H3N8 equine IVs, H3N8 and H3N2 canine IVs. Infrequent transmissions of animal IVs to humans result in isolated zoonotic infections; on very rare occasions, zoonotic IVs can adapt for efficient human-to-human transmission, initiate global pandemics, and continue to circulate in humans causing seasonal influenza disease [for recent reviews, see Krammer et al. (2018) and Liu et al. (2022)].

Interspecies transmission of IVs is usually accompanied by adaptive changes in their genomes required for improved viral fitness in the new host species. Adaptive changes in the receptor-binding properties of IVs and underlying amino acid substitutions in the attachment protein HA have been relatively well studied [reviewed by Matrosovich et al. (2006), Shi et al. (2014), Thompson and Paulson (2020), and Liu et al. (2023)]. Avian-to-human and avian-to-swine adaptation was shown to require a change in HA receptor-binding specificity from preferential recognition of Neu5Ac2–3Gal-terminated glycans (“avian-type” receptors) expressed on avian intestinal target cells to preferential recognition of Neu5Ac2–6Gal-terminated glycans (“human-type” receptors) expressed on target cells of airway epithelium in humans and pigs. Changes in receptor specificity were mediated by amino acid substitutions at the conserved positions of the avian HA, such as G228S and/or Q226L (the H3 numbering system is used throughout the text) in the case of H2N2/1957 and H3N2/1968 pandemic IVs and G225D/E and/or E190D (H1N1/1918 pandemic IV, H1N1 classical and avian-like swine IVs). Substitutions at some of these four “canonical” positions and/or several other HA positions of poultry-adapted IVs and sporadic mammalian isolates with H4, H5, H6, H7, H9, and H10 HAs have been found to facilitate binding to human-type receptors (Paulson and de Vries, 2013; Thompson and Paulson, 2020; Liu et al., 2022, 2023).

The primary function of IV neuraminidase is to attenuate HA interactions with decoy receptors [for reviews on structure and functions of IV NA, see von Itzstein (2007), Shtyrya et al. (2009), and McAuley et al. (2019)]. In the early stages of infection, NA removes sialic acid receptors from soluble sialylglycoproteins, mucous blanket, and the cellular glycocalyx, thereby facilitating virus motility and access to functional receptors on the cell membrane. In the late stages of infection, NA desialylates viral progeny and the surface of infected cells, preventing virus aggregation and promoting its release. As the receptor-destroying activity of NA counteracts the receptor-binding activity of HA, a balance of these activities with respect to the spectrum of sialoglycans present in the target host tissues is essential for IV fitness (Wagner et al., 2002; de Vries et al., 2020). However, in contrast to HA, there is much less understanding of NA specificity for Neu5Ac2–3Gal- and Neu5Ac2–6Gal-containing sialoglycans and of alterations in its substrate specificity during avian-to-mammalian adaptation of IVs.

Studies on the desialylation of soluble monovalent sialyloligosaccharides showed that N1 and N2 NAs of avian IVs predominantly hydrolyze Neu5Ac2–3Gal-terminated substrates, whereas NAs of human and porcine viruses have dual specificity with less efficient cleavage of Neu5Ac2–6Gal compared to Neu5Ac2–3Gal (Baum and Paulson, 1991; Couceiro and Baum, 1994; Kobasa et al., 1999; Mochalova et al., 2007; Gerlach et al., 2012; Garcia et al., 2013). The catalytic activity correlated with the binding avidity of the substrates to the NA, suggesting that the NA specificity for the avian-type and human-type sialoglycans depends on differences in their binding to the catalytic site (Mochalova et al., 2007; Garcia et al., 2013). Avian-origin N2 NA was introduced into the human population with the H2N2/1957 pandemic IV. Pandemic and early post-pandemic H2N2 virus strains had avian-type substrate specificity; the first detectable increase in their ability to cleave Neu5Ac2–6Gal was observed with A/England/12/1962 and correlated with the acquisition of the I275V amino acid substitution in the NA (Kobasa et al., 1999). The molecular mechanism behind the effect of this substitution on NA specificity remains undefined.

The NA of the H1N1/1918 pandemic IV differed from the closest avian N1 NAs by about 30 amino acid residues, including the Y347N substitution (the N2 numbering system is used throughout the text; position 347 corresponds to codon 344 of the N1 NA gene). This substitution was predicted to be a marker for mammalian adaptation (Reid et al., 2000) because it was also present in the N1 NA of the swine-adapted IV lineage that emerged from the independent introduction of an avian H1N1 virus into European swine in the late 1970s. In the crystal structures of N1 NA, the side chain of amino acid 347 protrudes into the solvent at the rim of the catalytic site; its main chain carbonyl oxygen forms a part of the Ca^2+^-binding site (Russell et al., 2006; Xu et al., 2008). Several research groups have found that the identity of amino acid at position 347 affects N1 NA avidity for the substrate, catalytic activity, and sensitivity to the NA inhibitors oseltamivir and zanamivir (Collins et al., 2009; Rameix-Welti et al., 2011; Yongkiettrakul et al., 2013; Dai et al., 2016; Su et al., 2021). Although these results demonstrated a contribution of amino acid 347 to substrate binding and catalysis, they were obtained using the non-natural substrate MUNANA and did not provide information on the effect of amino acid 347 on NA specificity toward Neu5Ac2–3Gal- and Neu5Ac2–6Gal-terminated sialoglycans.

X-ray analysis of NA binding to sialoglycans could not be performed for technical reasons. Therefore, molecular dynamics (MD) simulation was used to study the interactions of Neu5Ac2–3Gal- and Neu5Ac2–6Gal-containing trisaccharides with N1 NAs from H5N1 avian and H1N1 human IVs (Raab and Tvaroska, 2011; Jongkon and Sangma, 2012; Phanich et al., 2019). Collectively, these studies predicted that the catalytic sites of avian-type NAs favor binding to Neu5Ac2–3Gal- over Neu5Ac2–6Gal-containing substrates, whereas human-type NAs bind both substrates. Binding specificity was related, in part, to the poor fit of the Neu5Ac2–6Gal-containing glycan, in its bended solution-dominant conformation, into the catalytic site of the avian-type NAs. In contrast, the nearly linear conformers of the Neu5Ac2–3Gal-containing glycan were accommodated by both avian-type and human-type NAs. The amino acid at position 347 was predicted to contribute to binding specificity by steric hindrance and by interactions with the Neu5Ac and Gal moieties of the substrate. Because these modeling experiments used wild-type N1 NAs separated by multiple amino acid substitutions, further studies are needed to confirm and specify the predictions regarding the role of amino acid 347 in substrate specificity of N1 NA.

Here, we used molecular modeling to characterize complexes of two N1 NAs differing by the single substitution N347Y with two pentasaccharides representing models of the human-type and avian-type NA substrates. The NA-ligand complexes were built by molecular docking, and their geometry was refined by hundred-nanosecond MD simulation in explicit solvent. Using these models, we determined the effects of amino acid 347 on the conformation and atomic interactions of the ligand in the catalytic site, the free energy of binding, and the solvation of the glycosidic oxygen. In addition, we performed analysis of the host- and lineage-specific variation of the amino acid 347 among N1 NAs in the GISAID EpiFlu sequence database and assessed selective pressures at this position using comparable phylogenetic dN/dS techniques.

## 2 Materials and methods

### 2.1 Molecular modeling

#### 2.1.1 Models of sialoglycans and NAs

Two pentasaccharides, α-D-Neu5Ac(2 → 6)β-D-Gal(1 → 4)β-D-GlcNAc(1 → 3)β-D-Gal(1 → 4)β-D-Glc-OH, referred to in this study as 6S, and α-D-Neu5Ac(2 → 3)β-D-Gal(1 → 4)β-D-GlcNAc(1 → 3)β-D-Gal(1 → 4)β-D-Glc-OH (referred to as 3S) were used as models of the human-type and avian-type glycan receptors, respectively (Supplementary Figure S1). The pyranose ring conformations of 6S and 3S were set as chair ^2^C_5_ for Neu5Ac and as ^4^C_1_ for the remaining saccharides. The glycosidic backbone conformation of these glycans was defined by the set of dihedral angles ϕ_i_/ψ_i_, i = 1–4. The Neu5Ac2–3Gal linkage of 3S was defined by ϕ_1_ (C1-C2-O2-C3) and ψ_1_ (C2-O3-C3-H3). The Neu5Ac2–6Gal linkage of 6S was defined by ϕ_1_ (C1-C2-O6-C6), ψ_1_ (C2-O6-C6-C5), and ω (O6-C6-C5-H5). The other pairs of glycosidic dihedral angles were defined by four consecutive atoms H_i_-C_i_-O_i + 1_-C_i + 1_ and C_i_-O_i + 1_-C_i + 1_-H_i + 1_. The conformations of the glycosidic bonds were set according to the previously determined dominant unbound state conformations of the glycans in solution (Sassaki et al., 2013; Elli et al., 2021). The coordinate files of the two glycans were generated using the tleap application included in Ambertools 14.0 (Case et al., 2014). The crystal structure of the NA of the H1N1/1918 pandemic influenza virus A/Brevig Mission/1/1918 (3B7E) was obtained from the RCSB Protein Databank and designated NA/N in accordance with the nature of the amino acid at position 347. The N347Y point mutant of NA/N (designated NA/Y) was generated using PyMOL 2.4.0 (Schrödinger, LLC) with the default settings for the side chain rotational states.

#### 2.1.2 Molecular docking

The ligands, 3S and 6S, were docked into the catalytic sites of NA/Y and NA/N using Autodock 4.2 (Morris et al., 2009) to generate four complexes, 3S-NA/Y, 6S-NA/Y, 3S-NA/N, and 6S-NA/N. To perform the docking, we determined the Gasteiger charges (Gasteiger and Marsili, 1978) for the atoms of the glycans (ligands) and NAs (acceptors). The conformational sampling of 6S and 3S during docking was set to allow a full conformational exploration of the Neu5Ac-Gal linkages. In contrast, the inter-glycosidic dihedrals ϕ_2_/ψ_2_, ϕ_3_/ψ_3_ and the conformation of the whole disaccharide Gal1-4Glc were fixed in the docking simulations. Each docking simulation sampled 21 free rotational dihedral angles. The NAs were set as rigid macromolecules. The grid box dimensions were Lx = Ly = 80 points, Lz = 100 points (spacing Δx = Δy = Δz = 0.375 Å); the center of the grid box was set at the hydroxyl oxygen of Y406, the key catalytic residue occupying the central position at the base of the NA active site (Taylor and von Itzstein, 1994). In each simulation, the Lamarckian genetic algorithm search was performed with the following parameters: number of runs, 100; population size, 4,000; maximum number of energy evaluations, 3⋅10^7^; maximum number of generations, 300,000. The ligand-NA poses were selected for further geometry refinement by MD simulation based on the following criteria: (1) the position of the Neu5Ac residue in the center of the NA active site among the conserved residues R118, R292, R371, W178, D151, R152, and Y406; (2) the lowest predicted (Autodock 4.2) binding energy.

#### 2.1.3 MD simulation of the complexes 3S-NA/Y, 6S-NA/Y, 3S-NA/N, and 6S-NA/N

The four glycan-NA complexes obtained by docking were submitted to MD simulation in explicit solvent. Each complex was surrounded by a 15 Å-wide layer of TIP3P water molecules (Jorgensen et al., 1983) to form an orthogonal box (simulation cell) with edges of approximately 90 Å. The molecular mechanics force fields used, GLYCAM06 (Kirschner et al., 2008) and Amber (ff14SB) (Case et al., 2014), represented the “state of the art” force fields for glycans and proteins, respectively. Tleap (Ambertools 14.0) was used to build the topology and coordinate files of the complexes. The standard cut-off (12 Å) was applied to describe non-bonded electrostatic and dispersive interactions. Each simulation cell was minimized by running 100 K steps of the default minimization algorithm included in the NAMD 2.14 (Phillips et al., 2005). The software VMD 1.9.3 (Humphrey et al., 1996) was used for the MD simulation visualization and analysis. The number of particles (N), pressure (P), and temperature (T) were kept constant during the MD simulations. The constant simulation temperature (300 K) was maintained with a Lowe–Andersen thermostat; the Nosé–Hoover–Langevin piston algorithm controlled the pressure (1.01325 bar) applied to the cell walls. During the cell density equilibration steps (duration approximately 10–15 ns), a harmonic potential energy constraint (harmonic constant of 2.0 kcal mol^−1^) was applied to all atoms of the complexes, while water molecules were allowed to move freely. No additional constraints were applied at the equilibration and production stages of the MD simulation. For each simulated complex the equilibration period was estimated following the time evolution of two different properties, (1) the glycosidic dihedral angles ϕ_i_(t)/ψ_i_(t) (i = 1,4) of the glycan backbone; (2) the distance RMSD(t) of the glycan from its initial position (*t* = 0 ns); a stationary oscillatory behavior of these properties over time indicated the end of the equilibration and the beginning of the production MD simulation. During the MD simulation, all complexes were sampled every 10 ps for a period of 195 ns. The equilibration time was 110 ns for 6S-NA/N, 3S-NA/N and 3S-NA/Y, and 120 ns for 6S-NA/Y.

#### 2.1.4 Analysis of conformations of 6S and 3S

The conformational analysis of the 6S and 3S in the unbound state was performed previously (Elli et al., 2021), and the results are presented in this study with permission from the Biochemical Journal via the Copyright Clearance Center. The conformations of 6S and 3S bound to the catalytic site of NA/N and NA/Y were sampled by MD simulation, and the Ramachandran plots ϕ_i_(t)/ψ_i_(t) with corresponding density color maps for the four glycosidic linkages connecting the five sugar residues of the ligands were calculated. In these plots, the color gradient from blue to red is proportional to the density of states that were sampled by the MD simulations (from 110 or 120 ns to 195 ns). The Ramachandran plots, the 2D binning procedure and the density color maps were generated using the statistical software R (R Core Team, 2013), as described previously (Elli et al., 2021). The most populated states of the ω angle of 6S and of the internal dihedral angle C1-C2-C3-C4 of Neu5Ac (see Supplementary Figure S1) were determined at the production stage of the MD simulation using the 1D binning procedure implemented in the OriginPro 8.0 software (OriginLab Corp.). The internal dihedral angle C1-C2-C3-C4 was used to monitor the conformation of the Neu5Ac moiety. Values of this angle around 60° correspond to the chair ^2^C_5_ conformation typical for the terminal Neu5Ac residue in sialoglycans; values around ±180° characterize the distorted boat conformation observed in the crystal structures of NA complexes with the free Neu5Ac molecule (Varghese et al., 1992).

#### 2.1.5 Structure of the glycan-NA complexes

The structures of the NA complexes with 3S and 6S were determined by MD simulation as follows. First, the average structure of each glycan-NA complex was calculated for the production stage of the MD simulation using the Wordom 0.22-rc3 software (Seeber et al., 2007). Next, the MD snapshot having the smallest root mean square distance (RMSD) from the average structure of the complex was extracted from the MD simulation trajectory using VMD 1.9.3 software. Thus, the MD simulation snapshots taken at times 142.03, 150.44, 147.95, and 141.59 ns were selected to represent the complexes 6S-NA/N, 6S-NA/Y, 3S-NA/N, and 3S-NA/Y, respectively. These snapshots had RMSDs of 0.494, 0.511, 0.495, and 0.507 Å, respectively, from the corresponding average structures.

#### 2.1.6 Free energy of binding determined using MMPBSA approximation

To estimate the Poisson Boltzmann free energy of binding (*ΔG_PB_^bind^*) from the MD simulation trajectories of the glycan-NA complexes, we used Molecular Mechanics Poisson Boltzmann Surface Area (MMPBSA) method described previously (Weis et al., 2006; Elli et al., 2021). For each glycan-NA complex, the *ΔG_PB_^bind^* was determined as average on the production phase of MD simulation (between 130 to 195 ns) with a sampling frequency of 200 ps and a sample size of 325 poses covering a range of 65 ns. The standard error of the mean (SEM) of *ΔG_PB_^bind^* was calculated as σ/(N)^1/2^, where σ represents the estimated standard deviation, and N represents the number of samples. The contributions to *ΔG_PB_^bind^* of individual binding counterparts (saccharide and amino acid residues) (*ΔG_PB_^bind^*(i)) were calculated using the MMPBSA.py application (Miller et al., 2012) included in Ambertools 14.0 package. The chosen set of residues included all glycan residues (Neu5Ac, Gal, GlcNAc, Gal2, and Glc), and all amino acids of NA characterized by an energy cut-off |*ΔG_PB_^bind^*(i)| ≥ 0.3 Kcal mol^−1^. Negative and positive values of *ΔG_PB_^bind^(i)* reflect, respectively, favorable and unfavorable contributions of the residue to the binding energy.

#### 2.1.7 Distribution of the water molecules in proximity of the Neu5Ac-Gal glycosidic bond

The MD simulation in explicit solvent reproduces the distribution of water molecules at the interface between the complex (or the macromolecular surface) and the surrounding water (Raffaini et al., 2006; Raffaini and Ganazzoli, 2006). Here, we focused on the glycosidic oxygen of the Neu5Ac-Gal linkage, which is the target of hydrolysis catalyzed by NA (Taylor and von Itzstein, 1994; von Itzstein, 2007). The profile of the local concentration of the water molecules surrounding the oxygen atom was investigated using the radial pair distribution function *rdf(r)* (Levine et al., 2011) as implemented in VMD 1.9.3:


(1) $$rdf\left(r\right)=\lim_{dr\to 0}\left[\frac{V}{4\pi N_{\text{pair}}}\frac{p\left(r\right)}{r^{2}\;dr}\right]$$

where the variable *r* represents the distance between the glycosidic oxygen of the Neu5Ac-Gal bond (reference atom) and the oxygen atom of each H_2_O molecule, N_pair_ denotes the number of potential atom pairs in the system, and *V* is the volume of the simulation cell. *rdf(r) dr* reflects the concentration of water molecules in a spherical layer of infinitesimal thickness (between *r* and *r + dr*) centered on the glycosidic oxygen. *rdf(r)* profiles were calculated for each of the four simulated complexes sampled every 100 ps at the production stage (130 to 195 ns) of the MD simulation.

#### 2.1.8 Hydrogen bonds

Tight H-bonds were defined based on the following criteria: a distance less than 3 Å between the atoms of donor X and acceptor Y (X-H---:Y) and a corresponding angle (X-H---:Y) greater than 150° (Almond et al., 2006). These conditions were checked for all possible H-bonds between the glycan and the protein in four studied complexes at the production stage of MD simulation. The percentage of time H-bonds persisted during the simulation was estimated using Origin 8 software.

### 2.2 Analyses of NA sequences

#### 2.2.1 NA sequences, phylogenetic analyses, and amino acid prevalence at position 347

The sequences were obtained from the GISAID EpiFlu database (Shu and McCauley, 2017) accessed on June 19, 2023. Host- and lineage-specific nucleotide sequences were downloaded using the EpiFlu search filter “N1 NA, complete length” combined with each of the following filters: (1) “HA subtypes, H1-H4, H6-H16; Avian,” (2) “H5 HA; Avian,” (3) “H5 HA; Human,” (4) “H5 HA; Mammalian,” (5) “H1 HA; Swine,” (6) “Human; isolated before 2009,” and (7) “Human; isolated from 2009 to 2023.” Sequences were aligned using the MAFFT program implemented in the Unipro UGENE 47.0 (Okonechnikov et al., 2012). The datasets were processed using Bio-Edit 7.1.11 (Hall, 1999) and Jalview 2.11.2.7 (Waterhouse et al., 2009). Sequences with gaps, ambiguities, incomplete sequences, and sequences of laboratory-derived IVs were excluded; only one sequence from each cluster of identical sequences was retained. Due to the large number of H1N1pdm sequences (exceeding 30,000), a subset of 2,568 representative sequences was selected for the analyses. Maximum likelihood trees were generated using either the FastTree method (Price et al., 2010) implemented in Unipro UGENE or IQ-TREE 2 (Minh et al., 2020) with ModelFinder (Kalyaanamoorthy et al., 2017) and the ultrafast bootstrap approximation (Hoang et al., 2018). The trees were plotted using FigTree 1.4.4.^1^ Based on the trees, the sequences of swine IVs were separated into the classical and the avian-like lineage; the sequences of human IVs were separated into the A/H1N1/1918-like seasonal lineage and the A/H1N1/2009-like H1N1pdm lineage. In some of the sequence datasets, a few sequences failed to cluster with the rest. Some of those sequences were discarded as laboratory artifacts; the remaining atypical sequences represented either swine-virus-like isolates from birds and humans or human-virus-like isolates from pigs and birds; they were combined and analyzed separately. To facilitate analyses of the trees, we annotated the sequences with the single-letter code for the amino acid at position 347 (corresponds to codon 344 of the N1 NA ORF). The number of sequences in each group is listed in Table 3. The group name, virus name, and accession number for each analyzed sequence are shown on the global tree (Supplementary material, Global NA tree.nex and Global NA tree.svg). Prevalence of amino acids at position 347 was determined using the positional numerical summary tool of Bio-Edit.

To annotate titles of NA and/or HA sequences with specific amino acids of both proteins, we downloaded the sequences using the search filter “HxN1; HA + NA; complete length.” Curated HA and NA sequences were concatenated with MEGA11 (Tamura et al., 2021). Using Bio-Edit, we selected specific columns in the alignments of translated concatenated sequences and copied them into sequence titles.

#### 2.2.2 Selection pressure analyses

We applied several codon-based phylogenetic tests of natural selection to the global alignment of all N1 NA sequences [for a review see Spielman et al. (2019)]. These tests estimate the ratio of rates of non-synonymous and synonymous substitution (dN/dS) and compare it to the neutral expectation, dN/dS = 1 (FEL and MEME tests) (Kosakovsky Pond and Frost, 2005; Murrell et al., 2012). For analyses investigating within-group selection (e.g., within “Classical swine” group, or “H1N1pdm” group), we partitioned all branches in the combined NA tree using “conjunctive” labeling: each leaf of the tree has a label based on its annotation, and internal nodes (whose labels are not known) are assigned the same label as their descendants, but only when all of the descendants have the same label. The remaining internal nodes receive no label (“Unlabeled” group). This approach is conservative and will not attempt to infer virus group assignments for internal/ancestral nodes unless such assignment is unambiguous. Given G groups in the tree, we inferred G dN/dS ratios using maximum likelihood. For each group, we tested whether its dN/dS was different from 1, using the likelihood ratio test.

We also ran a multi-group MEME analysis, which allowed dN/dS to vary among branches within a group, and can be used to detect episodic selection, i.e., selection affecting only a proportion of branches in the tree (Murrell et al., 2012). For all dN/dS analyses, we used the HyPhy package (v 2.5.54).

## 3 Results

### 3.1 Molecular modeling

**Figure 1:**
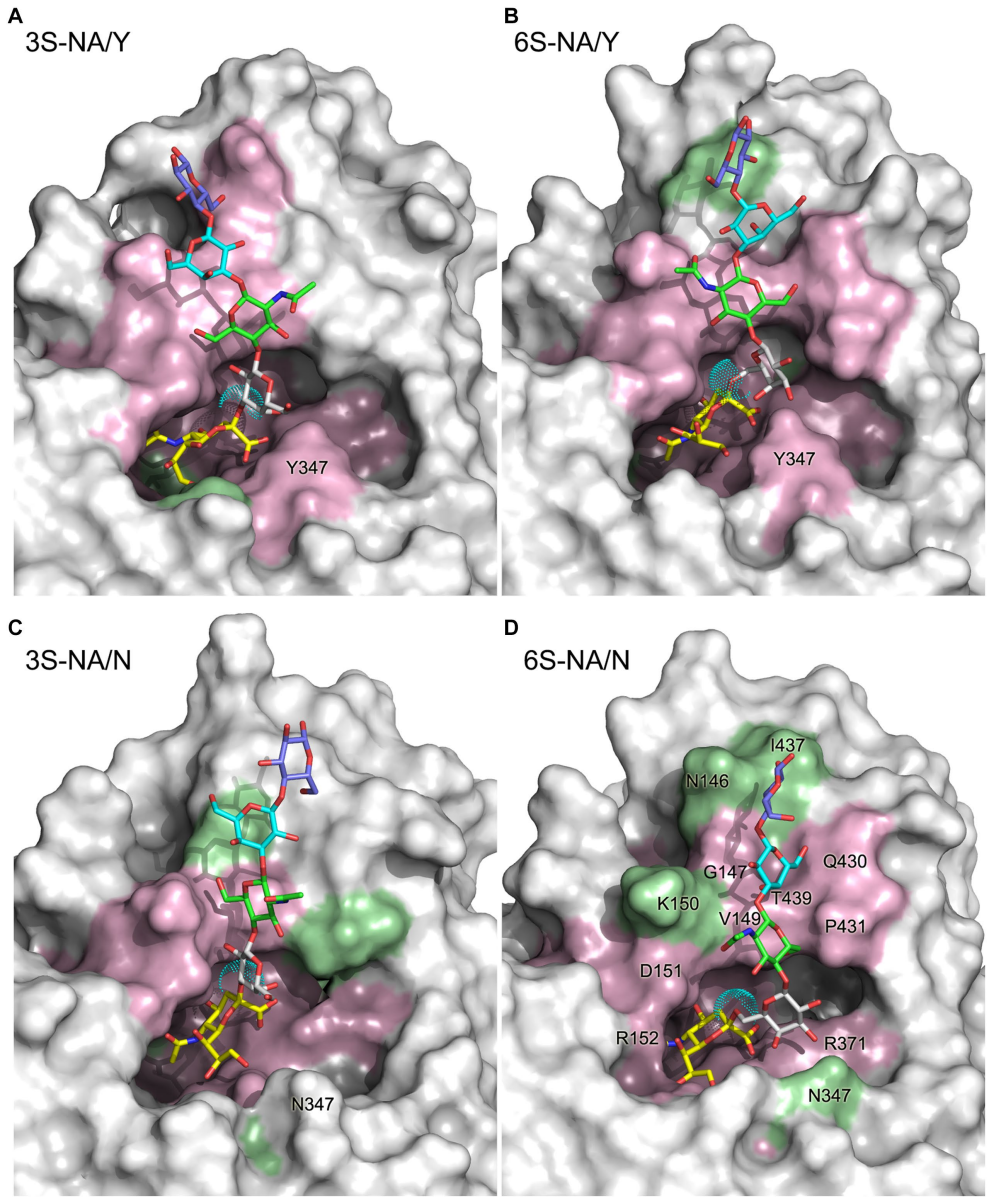
3D structure of the complexes 3S-NA/Y **(A)**, 6S-NA/Y **(B)**, 3S-NA/N **(C)**, and 6S-NA/N **(D)** predicted by MD simulation. The sialoglycans are shown as stick models with carbon atoms colored in yellow (Neu5Ac), white (Gal), green (GlcNAc), cyan (Gal2) and slate blue (Glc), and oxygen and nitrogen atoms colored in red and blue, respectively. Cyan dots show the van der Waals surface of the oxygen atom of the Neu5Ac-Gal linkage. The protein is presented as a molecular surface with atoms colored according to their contact distance to the sialoglycan (pink, < 4 Å; green, between 4 Å and 5 Å; white, >5 Å). Selected residues are labeled on the panel **(D)**.

To study recognition by the catalytic sites of N1 NAs of human-type and avian-type sialoglycans and to dissect the role of amino acid at position 347 in recognition, we modeled binding of the sialo-pentasaccharides 6S and 3S to the NA of human-adapted pandemic IV A/Brevig Mission/1/1918 (H1N1) and to its “avianized” N347Y mutant. Four complexes, 3S-NA/Y, 6S-NA/Y, 3S-NA/N, and 6S-NA/N, were built by docking, and their geometry was analyzed and refined by MD simulation in explicit solvent (Figure 1). The complexes 6S-NA/N and 3S-NA/Y were used to model interactions between human-type and avian-type NAs, respectively, with corresponding host-specific sialoglycans (referred to as “homologous” interactions throughout the text). The complexes 6S-NA/Y and 3S-NA/N modeled “heterologous” interactions.

#### 3.1.1 Alteration of the conformations of 6S and 3S upon binding to NA

**Figure 2:**
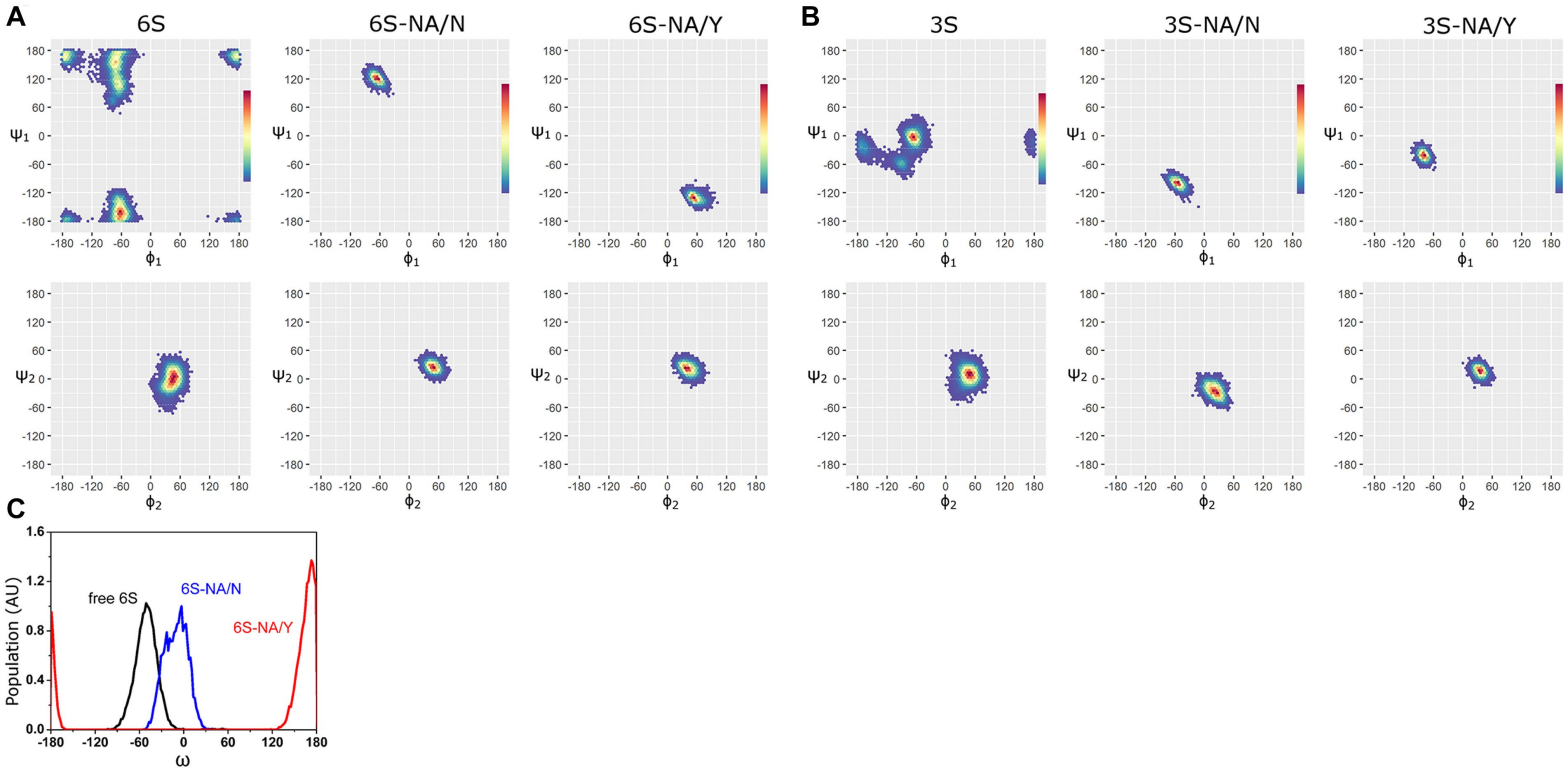
Ramachandran plots and color density maps of the glycosidic torsions ϕ_1_/ψ_1_ and ϕ_2_/ψ_2_ of the glycans 6S **(A)** and 3S **(B)** in unbound state [(Elli et al., 2021) with permission from Biochemical Journal] and in complex with NA/N and NA/Y. The color gradient from blue to red on each map is proportional to the increase in the population density of the states sampled by MD simulation. **(C)** Population (arbitrary units) of the ω angle of 6S in unbound state (black line) and in the complexes with NA/N (blue line) and NA/Y (red line).

The Ramachandran plots and the most populated conformations of the glycosidic torsion angles ϕ_i_/ψ_i_ for both free and bound sialoglycans sampled during the productive stage of MD simulations are presented in Figure 2, Supplementary Figure S2, and Supplementary Table S1. The ϕ_1_/ψ_1_ torsions of free 6S demonstrated significant conformational flexibility of the Neu5Ac2–6Gal linkage and populated four states, −62°/−163° (41%), −73°/155° (26%), −66°/110° (20%) and − 173°/170° (13%) (Elli et al., 2021) (see Figure 2A; Supplementary Table S1). In the homologous 6S-NA/N complex, the conformational space of the Neu5Ac2–6Gal linkage was reduced to one of these four states (−67°/122°). In contrast, none of the four states were observed in the heterologous 6S-NA/Y complex, in which case the Neu5Ac2–6Gal linkage adopted a new conformation (ϕ_1_/ψ_1_ = 52°/−130°) not previously seen in free Neu5Ac2–6Gal-terminated glycans or in their complexes with IV HA (Xu et al., 2009; Elli et al., 2014; Macchi et al., 2016). Distinctions in the recognition of Neu5Ac2–6Gal linkage by NA/N and NA/Y were also evident at the level of the linkage dihedral angle ω (O6-C6-C5-H5). More significant changes in ω (from *gauche* (−) to *trans*) occurred in the case of 6S binding to avian-type NA/Y compared to the homologous NA/N (Figure 2C).

MD simulations with the avian-type glycan 3S revealed analogous pattern. The Neu5Ac2–3Gal linkage of free 3S populated three states, −67°/−3° (74%), −92°/−58° (15%) and − 168°/−23° (11%) (Elli et al., 2021). The second conformation was selected when 3S bound to the homologous NA/Y (ϕ_1_/ψ_1_ = −79°/−42°), whereas binding to heterologous NA/N induced a new conformation (ϕ_1_/ψ_1_ = −55°/−100°) (Figure 2B; Supplementary Table S1).

In contrast to significant changes in the conformational space of the Neu5Ac-Gal linkage upon binding, much weaker effects were observed at the level of the Gal-GlcNAc linkage (ϕ_2_/ψ_2_) (Figure 2), and no effects were detected at the level of more distant saccharide residues (ϕ_3_/ψ_3_ of GlcNAc-Gal2 linkage and ϕ_4_/ψ_4_ of Gal2-Glc linkage, Supplementary Figure S2; Supplementary Table S1). This pattern suggests that the recognition of glycans by NA was primarily determined by the structure of the terminal Neu5Ac-Gal moieties.

These results demonstrated that the binding of sialoglycans to NA significantly reduced their conformational space. The extent of this effect (and the associated energy cost) depended on the interplay between the type of Neu5Ac-Gal linkage and the amino acid residue at position 347; the effect was less pronounced in the case of homologous glycan-NA interactions.

#### 3.1.2 Atomic contacts in the NA complexes with sialoglycans

**Figure 3:**
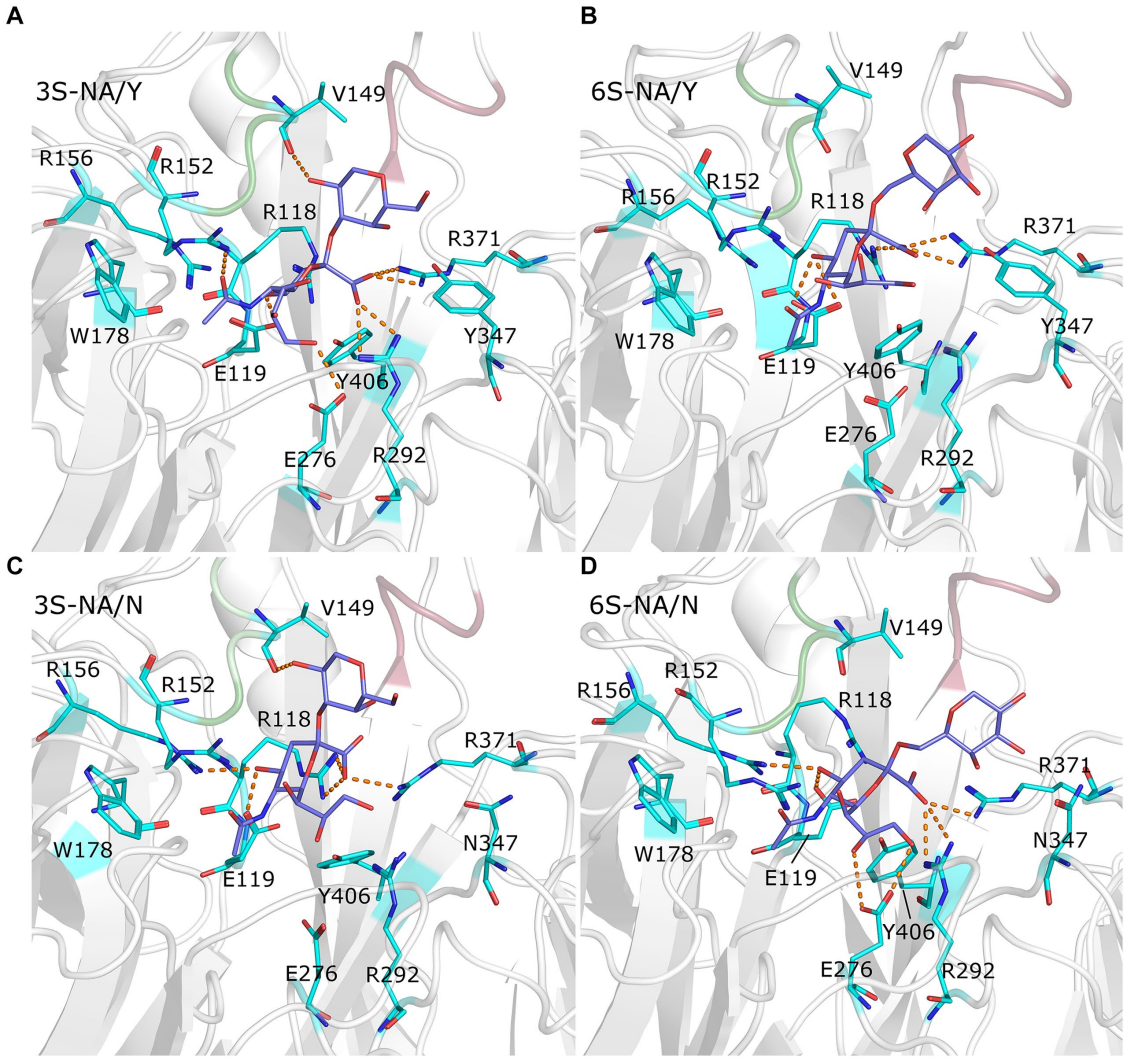
Close up view of the complexes 3S-NA/Y **(A)**, 6S-NA/Y **(B)**, 3S-NA/N **(C)**, and 6S-NA/N **(D)**. The white ribbon depicts the protein backbone, with loop 150 and loop 430 colored in green and pink, respectively. Stick models show terminal Neu5Ac-Gal moiety of the ligand (carbon atoms in slate blue) and selected contact residues (carbon atoms in cyan); oxygen and nitrogen atoms are colored in red and blue, respectively. The hydrogen bonds with the highest population are indicated by orange dashed lines.

The contact distances between sialoglycans and the protein in the complexes are color coded in Figure 1. Figure 3 illustrates the interactions between the terminal Neu5Ac-Gal moiety and the catalytic site of the NA. Selected contact distances, H-bonds and their persistence during the production phase of the MD simulation are presented in Table 1. In all four complexes, the Neu5Ac residue was located between the conserved amino acid residues R118, E119, D151, R152, R156, W178, R292, E276, R371, and Y406. However, the position of Neu5Ac with respect to the arginine triad (R118, R292, R371), residues R152, R156, and Y406, as well as loops 150 and 430, and contact distances between Neu5Ac and the protein differed between homologous and heterologous complexes.

**Table 1:** Selected contact distances (Å) in NA complexes determined in this study and in published crystal structures 2BAT, 1MWE, and 1 W21.

Contacting atoms (bold)		Complex						
NA	Glycan	3S-NA/Y	6S-NA/Y	3S-NA/N	6S-NA/N	2BAT	1MWE	1 W21
H-bonds and polar contacts								
R118 NC**N**H_2_	Neu5Ac C**OO-**	5.7	**2.8** (57)	**2.6** (86, 55)	4.8	**3.1**	**2.8**	**2.9**
R292 NC**N**H_2_	Neu5Ac C**OO-**	**2.6** (26, 53)	5.1	5.8	**3.1** (62, 28)	**3.2**	**3.3**	**3.3**
R371 NC**N**H_2_	Neu5Ac C**OO-**	**2.8** (21, 56)	**3.0** (67, 43)	**2.9** (86, 62)	**2.7** (94, 6)	**2.8**	**2.7**	**2.9**
E119 C**OO-**	Neu5Ac 4**O**H	**2.6** (67)	**3.0** (48, 15)	**2.6** (87)	**2.6** (92)	**3.3**	**3.3**	**3.1**
R152 NC**N**H_2_	Neu5Ac NHC**O**Me	**3.1** (49)	6.1	5.6	4.0 (6)	4.0	**2.6**	**2.9**
R156 NC**N**H_2_	Neu5Ac 4**O**H	3.9	**3.2** (10)	**2.9** (38)	3.8 (37)	4.8	4.7	4.7
E276 C**OO-**	Neu5Ac 8**O**H	4.7	5.5	4.9	**3.4** (95)	**2.6**	**2.7**	3.6
E276 C**OO-**	Neu5Ac 9**O**H	**3.0** (60)	4.4	7.0	**2.6** (89)	**3.1**	**3.4**	**2.7**
Y347 **O**H	Neu5Ac 9**O**H	7.4	4.2 (8)	-	-	-	-	-
N347 C**O**NH_2_	Neu5Ac 9**O**H	-	-	6.1	7.5	10.8	8.5	-
V149 C**O**	Gal 2**O**H	**2.9** (69)	-	**2.8** (58)	-	-	-	-
Q430 C**O**NH_2_	Gal2 6**O**H	6.3	6.1	7.6	3.8	-	-	-
K150 **N**Η_3_+	GlcNAc NHC**O**Me	12.4	3.6 (5)	7.8	6.2	-	-	-
Y347 **O**H	Gal 4**O**H	**3.0**	3.7	-	-	-	-	-
Y347 **O**H	Gal 3**O**H	-	**2.8**	-	-	-	-	-
N347 C**O**NH_2_	Gal 4**O**H	-	-	9.4	5.5	-	-	-
N347 C**O**NH_2_	Gal 3**O**H	-	-	10.9	5.9	-	-	-
van der Waals contacts								
Y406 **O**H	Neu5Ac **C2**	4.0	4.8	5.6	3.9	**3.2**	**3.2**	**3.1**
W178 **Cε3**	Neu5Ac NHCO**Me**	4.0	6.9	6.4	6.1	3.9	3.7	3.9
V149 CH**Me**_**2**_	GlcNAc **C3,C5,C6**	4.4 (C3)	5.1 (C3)	3.9 (C5)	4.4 (C6)	-	-	-
P431 **Cδ**	GlcNAc **C6**	7.3	5.3	10.5	4.0	-	-	-

The closest contacts (<3.5 Å) are shown in bold. Values in brackets depict percent of H-bond persistence (if higher than 5%). 2BAT, 1MWE, and 1 W21, complexes of free Neu5Ac with N2, N9, and N6 NAs, respectively (Varghese et al., 1992, 1997; Rudiño-Piñera et al., 2006).

The homologous complexes 3S-NA/Y and 6S-NA/N showed considerable parallelism in the NA interactions with the terminal Neu5Ac moiety of the glycan (Figures 3A,D; Table 1). Neu5Ac was co-planar with the arginine triad residues in both complexes. Tight H-bonds were formed by Neu5Ac with R292, R371, E119, E276 and either R152 (3S-NA/Y) or R156 (6S-NA/N). The distances between the hydroxyl group of the key nucleophilic residue Y406 and its target C2 atom of Neu5Ac (4.0 Å, 3S-NA/Y; 3.9 Å, 6S-NA/N) were compatible with the proposed role of Y406 in NA-mediated hydrolysis (von Itzstein, 2007; Vavricka et al., 2013). Comparison of 3S-NA/Y and 6S-NA/N with the published crystal structures of NA complexes with free Neu5Ac revealed a marked similarity in the contact distances of the Neu5Ac residue in these complexes (Table 1). This finding suggests that, in the homologous complexes, the asialic moieties of the bound glycan do not interfere with the optimal position of the terminal Neu5Ac moiety in the NA catalytic site. Importantly, the Gal residues of the glycan in the complexes 3S-NA/Y and 6S-NA/N facilitated binding through the formation of polar contacts and weak transient H-bonds with the side chain of amino acid 347. Thus, during the MD simulation, the 4-hydroxyl of Gal of 3S oscillated within 3.0–3.5 Å from the hydroxyl group of Y347, whereas the 4-hydroxyl of Gal of 6S oscillated within 4.7–5.9 Å from the side chain of the carboxamido group of N347. In addition to interacting with amino acid 347, the asialic residues of 3S and 6S also interacted with V149. Gal made polar contacts with V149 in the 3S-NA/Y complex, whereas GlcNAc was involved in hydrophobic interactions with this amino in the 6S-NA/N complex. Interestingly, in 6S-NA/N, the GlcNAc and Gal2 residues of the glycan approached loop 430 and were engaged in polar and hydrophobic interactions with Q430 and P431, respectively (Figure 1D; Table 1). This finding supports the previous hypothesis about potential role of amino acid 430 in the substrate specificity of human-type NAs (Jongkon and Sangma, 2012).

The heterologous complexes 6S-NA/Y and 3S-NA/N shared a few structural features that differentiated them from both the homologous complexes and the published Neu5Ac-NA co-crystal structures. Thus, 6S bound to NA/Y in a tilted orientation of the Neu5Ac residue with respect to the arginine triad. Compared to the 3S-NA/Y complex, the Neu5Ac moiety of 6S moved away from loop 340 toward loop 150, lost H-bonds with R292 (distance, 5.1 Å), R152 (6.1 Å) and E276 (4.4 Å), and acquired tight H-bonds with R118 (2.8 Å) and R156 (3.2 Å) (Figures 1B, 3B; Table 1). The distance between the hydroxyl group of Y406 and the C2 carbon atom of Neu5Ac has increased to 4.8 Å in the 6S-NA/Y complex, compared to 4.0 Å in the 3S-NA/Y complex. Observed differences in the binding of 3S and 6S to NA/Y were likely caused by a larger footprint of the Neu5Ac2–6Gal moiety, a steric conflict of the 6-linked Gal residue with Y347 (see the pink surface in Figure 1B), and resolution of this conflict by the shift of the sialoglycan toward loop 150.

Similar to the binding of 6S to NA/Y, 3S bound to NA/N in a tilted orientation characterized by the absence of H-bonds between Neu5Ac and R292, R152 and E276 and by the formation of H-bonds with R118, R371 and R156 (Table 1). Due to this orientation, the Neu5Ac-Gal moiety shifted toward loop 150, and the distance between Y406 and the target C2 atom of Neu5Ac increased to 5.6 Å. The tilted orientation of the Neu5Ac-Gal moiety in 3S-NA/N was stabilized by the H-bond and hydrophobic interactions of Gal with V149 (Figure 3C; Table 1). The close contact between the ligand and loop 150 in 3S-NA/N and 6S-NA/Y is illustrated in the Figures 1B,C by the dotted van der Waals spheres of glycosidic oxygen atoms approaching this loop.

In summary, these results suggested that N347 and Y347 bind the homologous sialoglycans 6S and 3S, respectively, with an almost identical optimal orientation of the sialic acid moiety with respect to the contact residues of the catalytic site. This orientation depends, at least in part, on van der Waals and polar interactions of amino acid 347 with the Gal residue of the glycan.

#### 3.1.3 Poisson-Boltzmann free energy of binding

**Figure 4:**
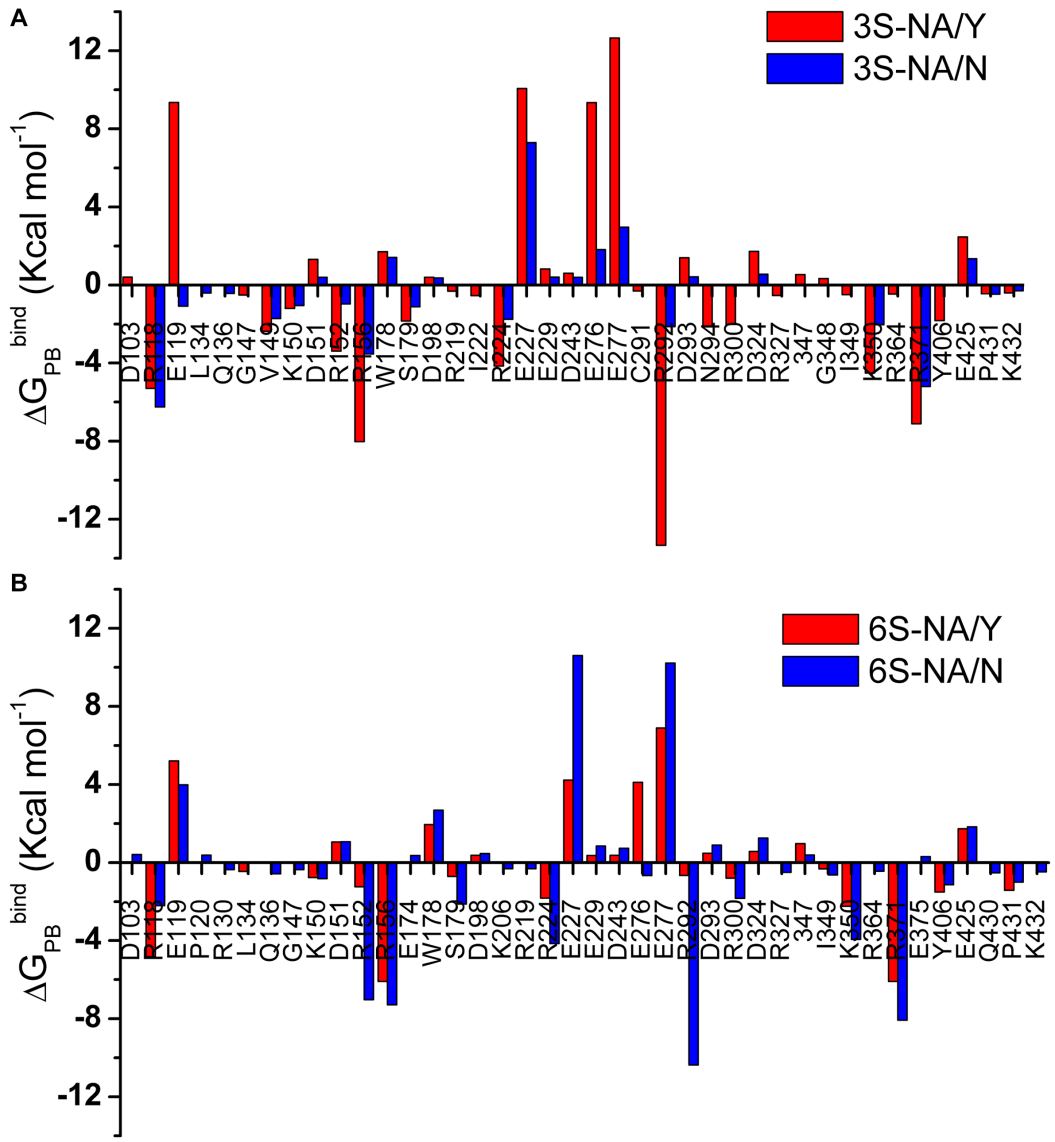
Per amino acid residue decomposition of *ΔG_PB_^bind^* for the complexes of 3S **(A)** and 6S **(B)** with NA/Y (red) and NA/N (blue). Only residues with absolute values of *ΔG_PB_^bind^* greater than 0.3 Kcal mol^−1^ are shown.

The Poisson Boltzmann free energy of binding *ΔG_PB_^bind^* (Weis et al., 2006) was calculated from the MD trajectories of glycan-NA complexes and decomposed into the contributions of individual saccharide residues of the glycan (Table 2) and amino acid residues of the protein (Figure 4). It should be noted that the MMPBSA procedure used to calculate *ΔG_PB_^bind^* does not account for the conformational changes (and associated energy costs) that the ligand and receptor undergo upon binding.

**Table 2:** Total *ΔG_PB_^bind^* (±SEM) of the complexes and contributions of saccharide residues (Kcal mol^−1^).

*ΔG_PB_^bind^* component	Complex			
	3S-NA/Y	6S-NA/Y	3S-NA/N	6S-NA/N
Total	−26.8 (±0.3)	−18.7 (±0.3)	−30.7 (±0.4)	−37.9 (±0.4)
Neu5Ac	−11.5	−10.5	−9.5	−16.8
Gal	1.1	0.9	−0.5	1.7
GlcNAc	0.3	1.5	0.0	2.1
Gal2	−0.4	0.0	−0.4	2.1
Glc	0.9	−0.1	−0.3	0.2

Based on the values of total *ΔG_PB_^bind^*, the human-type NA/N bound 6S with a higher avidity in comparison to 3S, whereas avianized NA/Y bound 3S more strongly than 6S. The N347Y substitution markedly reduced NA binding to 6S (*ΔG_PB_^bind^* difference, 19.2 Kcal mol^−1^) and had weak unfavorable effect on binding to 3S (3.9 Kcal mol^−1^). This pattern generally agreed with the results of MD simulation performed using sialotrisaccharides and NAs from wild type human and avian IVs (Phanich et al., 2019). Neu5Ac provided the major favorable contribution to the binding energy among the saccharide residues of the ligand, with the highest contribution in the case of 6S-NA/N complex (−16.8 Kcal mol^−1^). The other residues, including penultimate Gal, made insignificant and frequently unfavorable contributions in all complexes (Table 2).

The amino acid residues with charged side chains made the highest favorable and unfavorable contributions to *ΔG_PB_^bind^* (Figure 4), highlighting the key role of electrostatic forces in the binding. The arginine triad R118, R292 and R371 together with R152, R156, and R224 had the most significant favorable effect on binding. A few amino acids without charged side chains, including S179, Y/N347 and Y406, also favorably contributed to binding avidity. In contrast, residues with the side chain carboxylic groups (E119, E227, E276, E277) destabilized the glycan-NA complexes. Interestingly, loop 150 containing positively charged R152 and R156 had a much higher favorable energy share compared to loop 430 (Q430, P431, K432). In most cases, both favorable and unfavorable effects were more pronounced in the homologous complex than in the heterologous complex of the same sialoglycan (as shown by the data in the same panels of Figure 4). However, R118 deviated from this pattern, suggesting a potential unique function for this residue in recognition. Of note, V149 contributed favorably to the binding in both 3S complexes as a result of its hydrophobic interactions with Gal (Table 1).

In summary, estimation of the free energy of binding reveals that avian-type NA/Y binds human-type 6S significantly weaker than the homologous 3S and that substitution Y347N markedly improves binding of 6S and slightly increases binding of 3S. Decomposition data show that this pattern does not correlate with the direct contribution of amino acid 347 to *ΔG_PB_^bind^* but primarily depends on the effect of this amino acid on favorable electrostatic interactions of 3S and 6S with the arginine triad, E119, E276, and other conserved residues.

#### 3.1.4 Distortion of the Neu5Ac moiety in the complexes with NA

**Figure 5:**
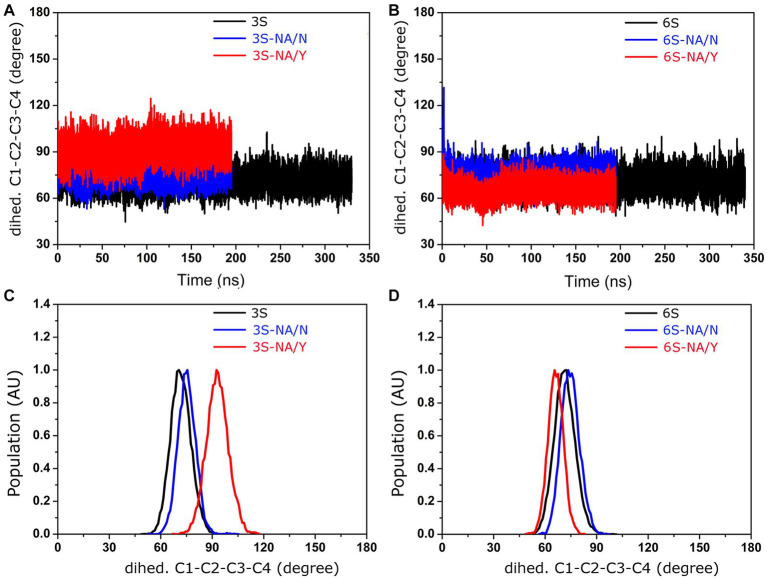
Conformation of the dihedral angle C1-C2-C3-C4 of the Neu5Ac residue of 3S and 6S in solution (black) and in complexes with NA/Y (red) and NA/N (blue). **(A,B)** MD simulation trajectories. **(C,D)** Population (arbitrary units).

The initial stages of the proposed mechanism of NA-mediated hydrolysis involve the binding of sialoglycan with its Neu5Ac moiety in the chair ^2^C_5_ conformation (with the carboxylate in the axial position), followed by the distortion of the Neu5Ac ring to a pseudo-boat conformation (with the carboxylate in the pseudo equatorial position). The distortion is mediated by the ionic, hydrogen bond, and steric interactions of Neu5Ac with active site residues; it induces formation of a strained oxocarbonium ion, ultimately resulting in the cleavage of the glycosidic bond (Janakiraman et al., 1994; Taylor and von Itzstein, 1994). Taking this mechanism into account, we decided to examine evolution of the Neu5Ac ring conformation during interaction of 3S and 6S with the NAs (Figure 5). MD simulation of the 3S-NA/Y complex revealed substantial alteration of the internal dihedral angle C1-C2-C3-C4 of Neu5Ac (mean value 93°) with respect to its conformation in the free ligand (mean value 72^o^) indicative of ring distortion toward pseudo-boat. Minimal distortion (if any) was observed in the case of the 3S-NA/N complex (Figures 5A,C). In the case of 6S, there was a slightly greater Neu5Ac distortion in the 6S-NA/N complex compared to 6S-NA/Y complex (Figures 5B,D), although the effect was smaller than that observed in 3S-NA/Y.

#### 3.1.5 Solvation of the glycosidic oxygen of Neu5Ac-Gal linkage

**Figure 6:**
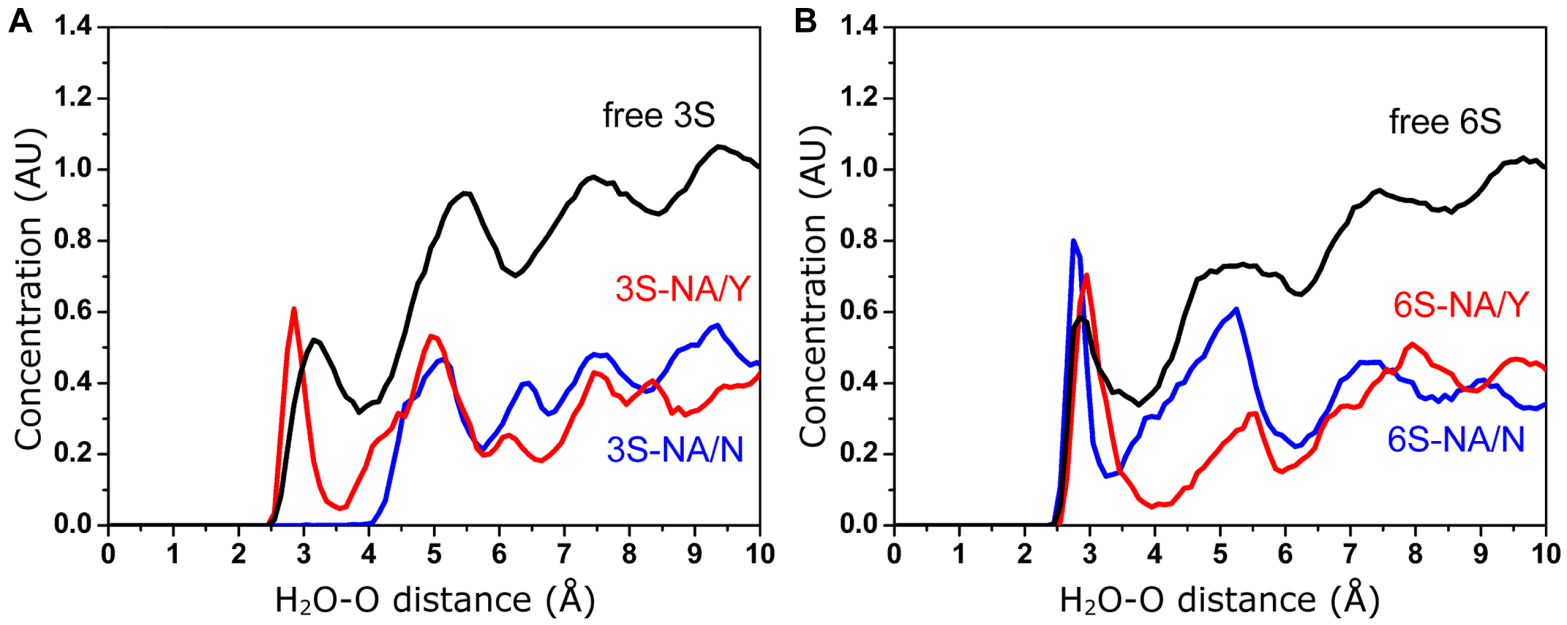
Concentration (arbitrary units) of water molecules in the vicinity of the glycosidic oxygen of the Neu5Ac-Gal linkage determined by MD simulation. **(A)** Free and bound 3S. **(B)** Free and bound 6S.

According to the proposed solvent-assisted mechanism of hydrolysis catalyzed by NAs, water must be present in the catalytic pocket. In particular, the water molecule located near D151 and R152 may participate in the NA-mediated proton transfer to the glycosidic oxygen atom of the substrate (Chong et al., 1992; Taylor and von Itzstein, 1994). Thus, the efficiency of hydrolysis may be affected by the concentration of water molecules surrounding the Neu5Ac-Gal linkage. As the MD simulation in explicit solvent allows the analysis of water molecules at the interface between the complex and the solvent, we calculated the distribution profile of water around the glycosidic oxygen in both free 3S and 6S, as well as their complexes with NA/Y and NA/N as described in the Methods section (Figure 6). In the case of free glycans, local maxima of water concentration were observed at 3.2, 5.5, and 7.4 Å (3S) and 2.8, 5.3, and 7.5 Å (6S). These maxima represented the first, second and third hydration shells, respectively, surrounding the target glycosidic oxygen. The concentrations of water molecules in the second and the third shells decreased in all complexes as compared to free 3S and 6S. Moreover, the first three peaks were shifted in the 3S-NA/N complex toward longer distances indicating a significant depletion of water in this complex in comparison to the homologous 3S-NA/Y complex (Figure 6A). Similarly, a greater depletion of water was observed in the complex 6S-NA/Y compared to its homologous counterpart 6S-NA/N (Figure 6B). These effects correlate with the observed shift of the Neu5Ac-Gal moieties in heterologous complexes 6S-NA/Y and 3S-NA/N toward loop 150 and the accompanying shielding of the glycosidic oxygen from the solvent (see models in the Figure 1).

### 3.2 Analysis of NA sequences

#### 3.2.1 Prevalence of amino acid 347 in avian and mammalian IV lineages

**Figure 7:**
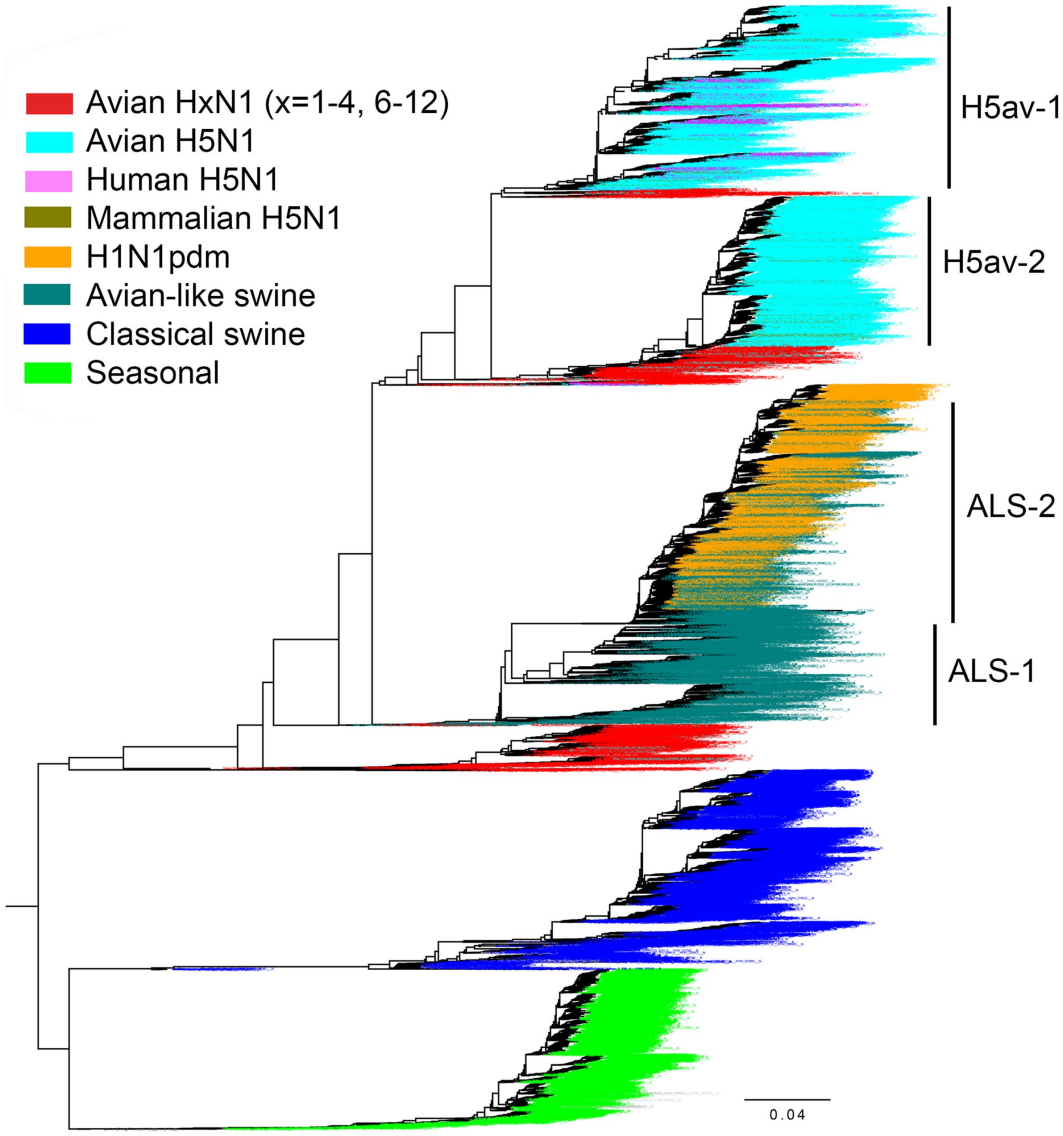
Phylogenetic relationship of 18,097 N1 NA gene sequences from the GISAID EpiFlu database used in this study. For the H1N1pdm lineage, only 2,568 representative sequences were included in the tree. The colors of the taxa names correspond to the virus group as indicated in the legend. Group separation of avian H5N1 IVs and avian-like swine IVs is illustrated. The tree was generated with FastTree. The fully annotated tree in nexus and svg formats can be found in the Supplementary information.

To investigate the prevalence of amino acid 347 in N1 NAs in different host species, we analyzed sequences retrieved from the GISAID EpiFlu database (Figure 7). In agreement with previous reports (Xu et al., 2012; Hufnagel et al., 2023; Yang et al., 2023), the N1 NAs were represented by the following major groups and lineages. No HxN1 sequences with HA subtypes H13-H16 were found in the database. The NAs of avian IVs with HA subtypes H1-H4 and H6-H12 were located near the root of the global phylogenetic tree and shared a common ancestor with two H1N1 mammalian lineages. Both these lineages, the “seasonal” human IVs and the “classical” swine IVs, were derived from the 1918 influenza pandemic. The second circulating swine IV lineage, commonly called “avian-like swine” (ALS) lineage, originated from an H1N1 avian ancestor; the first ALS viruses were isolated in Belgium and Germany in 1979. The 2009 influenza pandemic was initiated by a reassortant swine virus (H1N1pdm) that contained the NA gene from the ALS lineage. The novel H1N1pdm lineage replaced the previous seasonal human IVs. The H1N1pdm viruses were repeatedly reintroduced from humans to pigs, and this phenomenon significantly influenced the evolution of the ALS lineage (Hufnagel et al., 2023). Therefore, we divided the ALS sequences into two groups as shown in Figure 7. The sequences of highly pathogenic and panzootic H5N1 avian IVs comprised the last major N1 NA group. Due to the importance of H5N1 viruses for animal and human health, their sequences were significantly overrepresented compared to other avian sequences. We divided the NAs of all H5N1 IVs into four groups containing sequences from human isolates (H5hum), sequences from other mammalian isolates (H5mam), and two groups of avian sequences (Figure 7). The H5av-1 group included NAs of the A/goose/Guangdong/1996-like viruses from 1997–2023 (Gs/Gd-like lineage). The H5av-2 group included NAs of clade 2.3.4.4b H5N1 IVs that emerged in 2020, spread globally, and have been causing large outbreaks of infection in wild birds, poultry and mammals in 2020–2023 (Kandeil et al., 2023; Tian et al., 2023). Both H5av groups also included a small number of non-zoonotic H5N1 IVs from wild birds and poultry.

The number of NA sequences in each group and the prevalence of amino acid 347 are shown in Table 3. Our initial analysis of avian NAs revealed an unusually high frequency of substitutions at position 347 in H6N1 samples. Therefore, we decided to analyze H6N1 viruses separately. Y347 was perfectly conserved in the NAs of avian viruses with HA subtypes H1-H4 and H7-H12. The H6N1 group contained 11.6% of NA variants with the Y347F substitution. The H5av-1 group contained 1.33% of mutant NA sequences, ten times more than the H5av-2 group. It remains unclear whether this difference reflects a shorter evolutionary time of the H5av-2 lineage, unique virus properties, or a combination both.

**Table 3:** Prevalence of amino acids at position 347 of N1 NA.

Virus group	SeqN	N	D	Y	F	H	K	Q	S	T	G	%
Avian HxN1 (x = 1–4, 7–12)	918			918								0.00
Avian H6N1	464			410	54							**12.6**
H5av-1	2,627		15	2,592	1	17		1	1			**1.33**
H5av-2	2,315			2,312		3						0.13
H5hum	334			332		1					1	0.60
H5mam	186			185		1						0.54
ALS-1	1,588	1,572	2	14								**1.07**
ALS-2	1,269	1,262	1			1	5					0.55
Classical swine	3,174	3,167	7									0.22
Seasonal	2,551	1,728	823									**32.3**
H1N1pdm, all	32,752	32,723	12	1		2	11		2	1		0.089
H1N1pdm, representative	2,568	2,566	1				1					0.078

SeqN, number of sequences analyzed; %, percentage of amino acids other than Y in avian IVs and other than N in human and swine IVs (percentages greater than 1 are shown in bold).

Among the human and porcine IV NAs, only one H1N1pdm sequence (0.09%) and 14 ALS-1 sequences (1.1%) contained the avian-type 347Y. NAs from classical swine, ALS, and H1N1pdm lineages contained almost exclusively N347, whereas NAs from seasonal human IVs contained either N347 or D347.

The NAs of 101 atypical sporadic swine-virus-like isolates from humans and birds and human-virus-like isolates from pigs as a rule contained the same residue 347 as their closely related original host species (see sequences labeled “Atypical” in the tree in Supplementary material).

#### 3.2.2 Evolution of position 347 in seasonal and ALS-1 lineages

**Figure 8:**
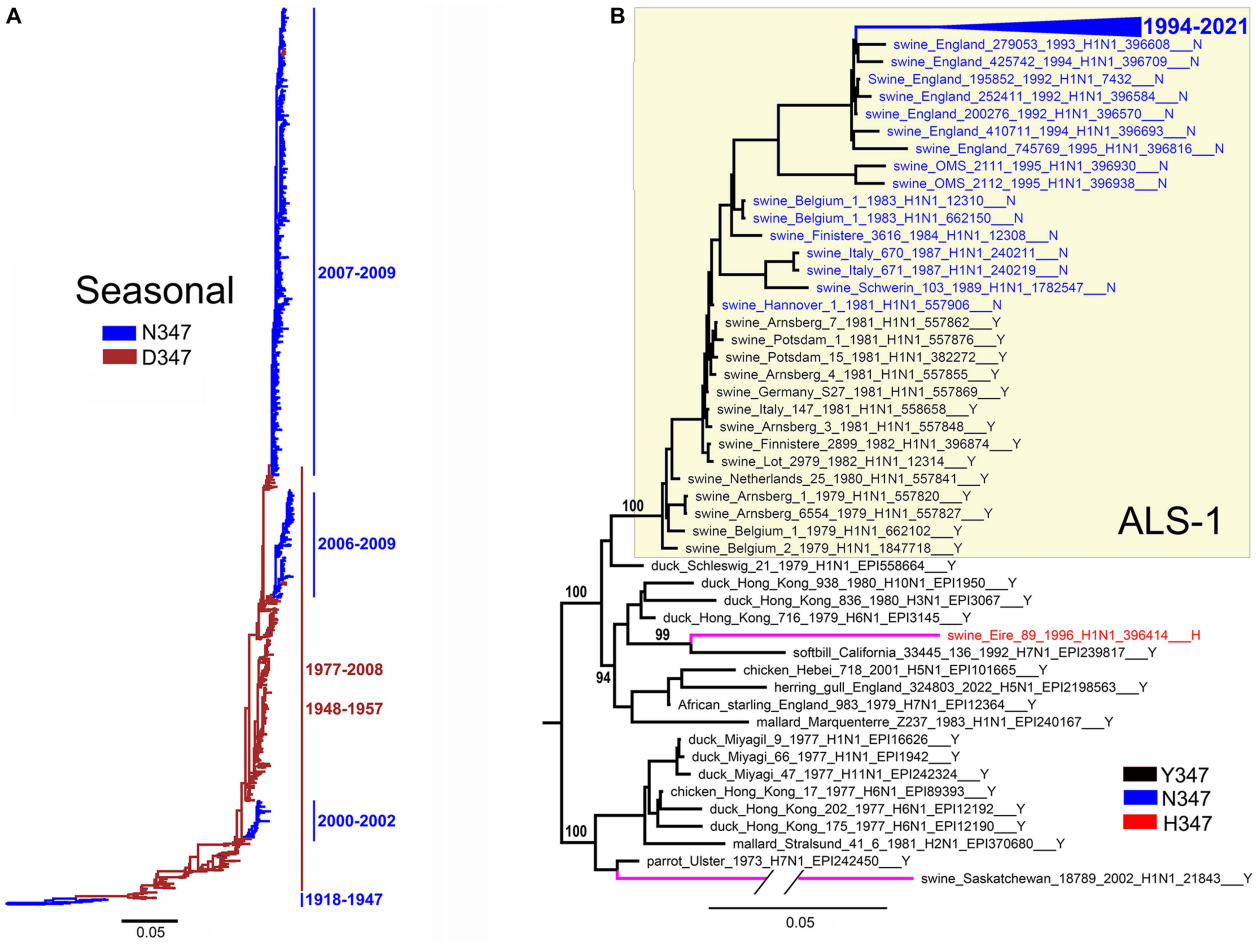
Substitutions at position 347 color-coded on NA phylogenetic trees as indicated in the panel legends. **(A)** Seasonal human lineage. Taxa names are not shown. Bars and numbers on the right present circulation periods of NA clades with N347 (blue) and D347 (brown). **(B)** The tree displays ALS-1 lineage (yellow box), phylogenetically close avian IVs, and two independent avian-like porcine isolates indicated by magenta branches. The branch containing sequences of IVs isolated after 1994 is collapsed for clarity (blue triangle). Numbers on specific branches represent bootstrap support values.

**Figure 9:**
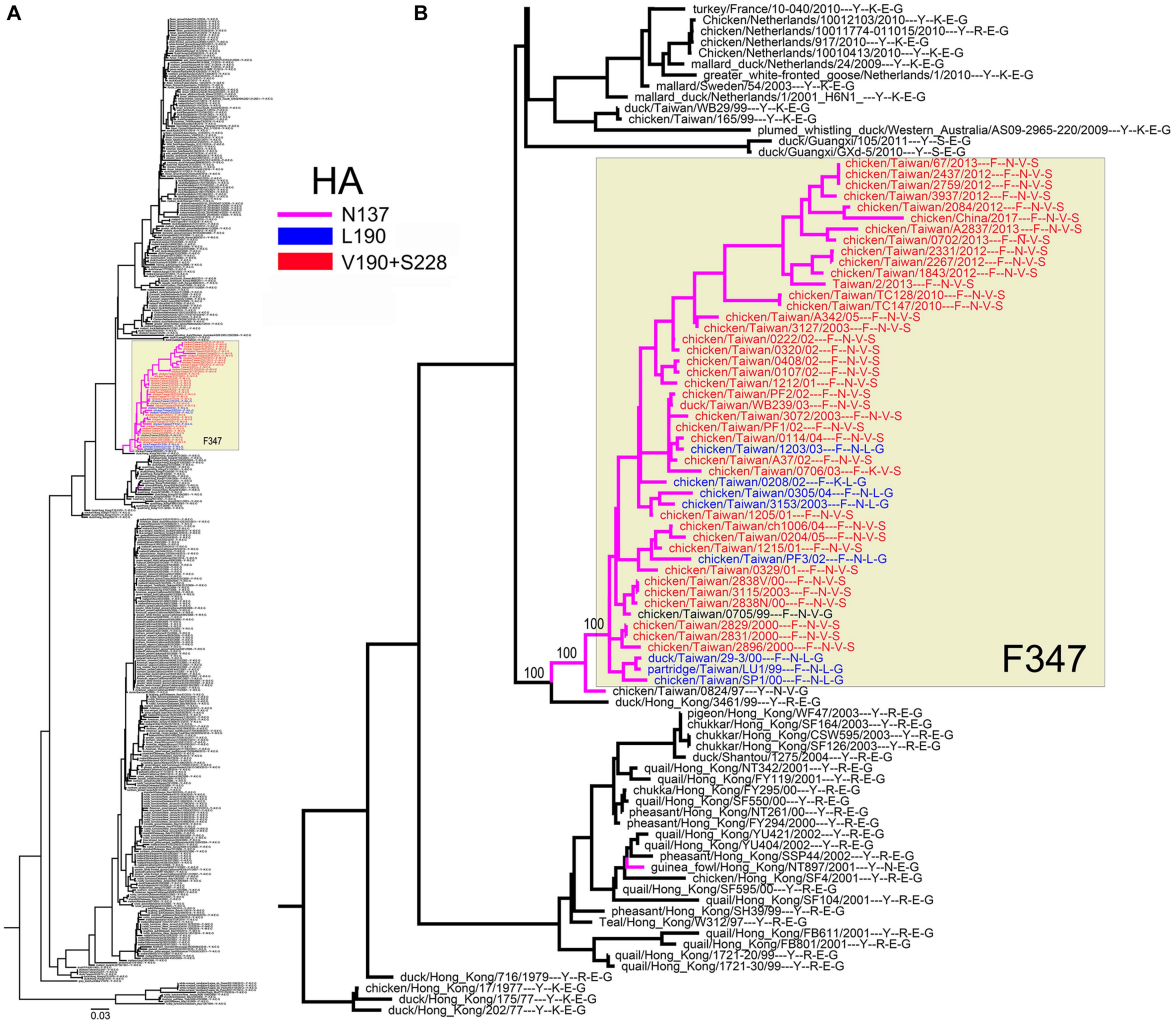
Association of the Y347F substitution in H6N1 NA with amino acids at HA positions 137, 190, and 228. **(A)** Phylogenetic relationships of NA. Colors denote the NA clade with F347 (yellow box) and the HAs with N137 (purple branches), 190 L (blue taxa names), and 190 V + 228S (red taxa names). **(B)** Enlarged partial view with specific amino acid residues listed after the strain name. The first character defines the NA residue 347, the next 3 characters define the HA residues 137, 190, and 228. Numbers on specific branches represent bootstrap support values.

**Figure 10:**
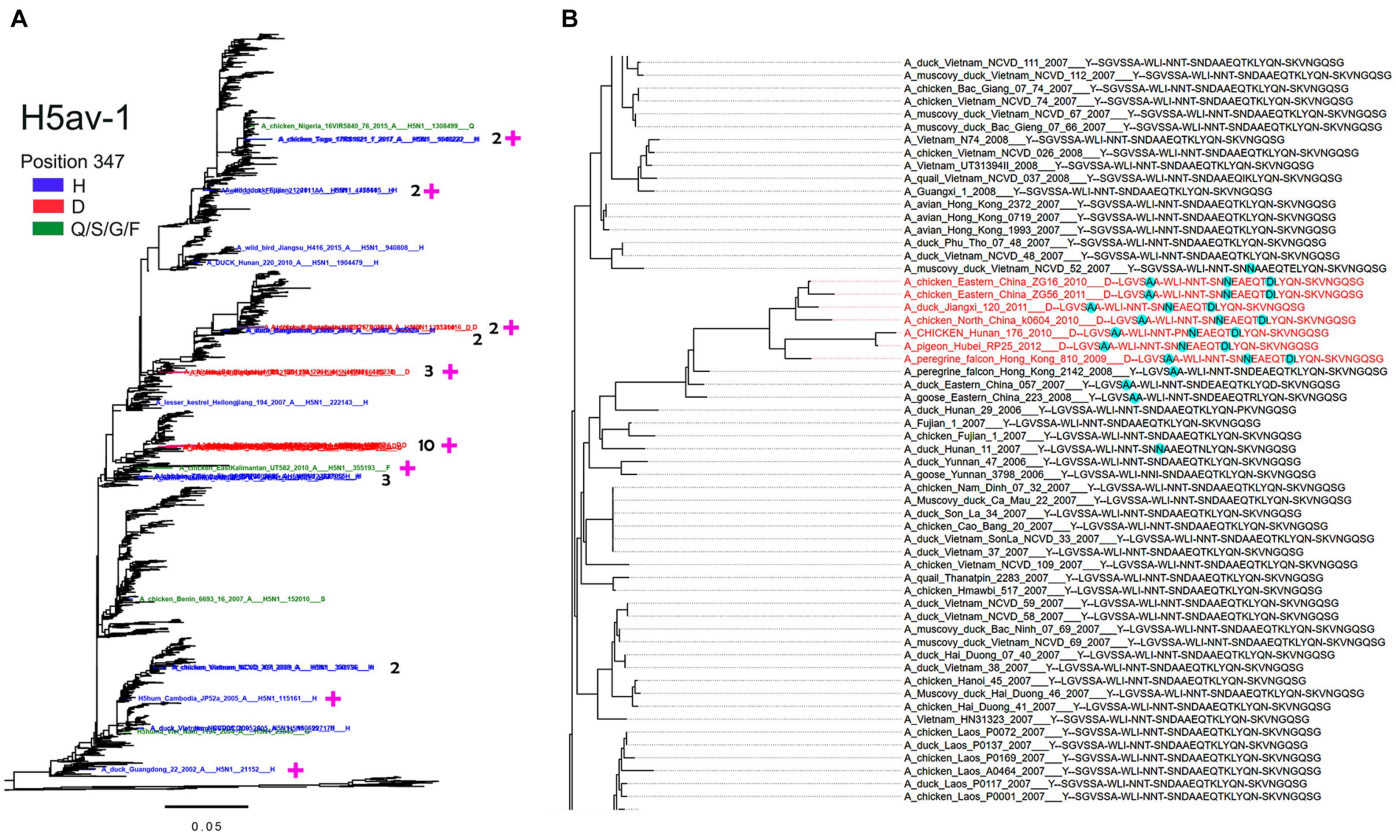
Association of substitutions at position 347 in H5av-1 NA with substitutions in HA. **(A)** H5av-1 NA tree rooted to A/chicken/Scotland/1959. The names of NAs with Y347 are not displayed. Taxa with the NA substitution 347 are colored as shown in the legend. For clusters of 2, 3, and 10 mutant sequences, the cluster size is indicated. Magenta plus signs mark sequences with associated substitutions in the HA. **(B)** A section of the H5 HA tree with the cluster of Y347D mutants colored in red. The taxa names, such as A_duck_Jiangxi_120_2011___D--LGVSAA-WLI-NNT-SNNEAEQTDLYQN-SKVNGQSG, include the virus name followed by NA residue 347 and groups of HA residues 133–138, 153–155, 158–160, 185–197, 221–228 separated by minus sign. Cyan highlights amino acid residues that distinguish corresponding HAs from others in the tree section. The full tree in nexus and svg formats can be found in the Supplementary material.

For the NA groups containing more than 1% of position-347 variants, we studied the location of these variants on the phylogenetic tree (Figures 8–10 and Global tree.nex/Global tree.svg in Supplementary material).

The H1N1/1918-derived seasonal human IVs circulated in 1918–1957 and 1977–2009 (Krammer et al., 2018). The NAs of the 1918 virus and its successors isolated between 1933 and 1947 contained N347 (Figure 8A). The N347D substitution occurred around 1947, and viruses with this substitution circulated in 1948–1957 and 1977–2008. During this time, the IVs with the D347N reversion emerged on three separate occasions and circulated together with the D347 variants in 2000–2002 and 2006–2009. These observations suggest that both N347 and D347 support the replication of seasonal viruses in humans and that occasional swapping of N and D occurs, conferring epidemiological advantage to the novel variant and leading to its expansion.

All 14 NAs of the ALS-1 lineage containing the avian-type Y347 were located at the root of the avian-like lineage (Figure 8B). The Y347N substitution occurred in 1981 and was strictly maintained thereafter. The only other substitution in the ALS-1 NAs, N347D (Table 3), was present in a cluster of two viruses isolated in 2013 (see Global tree in Supplementary material). In addition to the stable ALS lineage, two atypical H1N1 IVs, A/swine/Eire/1996 and A/swine/Saskatchewan/2002, represented independent introductions of avian IVs into swine (Figure 8B; Karasin et al., 2004; Lycett et al., 2012). Interestingly, these IVs contained canonical markers of HA adaptation to Neu5Ac2-6-Gal-terminated receptors, the E190D substitution (both viruses) and the Q226L substitution (A/swine/Eire/1996). The latter virus also had the Y347H substitution in NA.

#### 3.2.3 Substitutions in the NA of H6N1 and H5N1 viruses are associated with substitutions in HA RBS

All H6N1 viruses with the Y347F NA substitution belonged to the specific clade of poultry IVs from Taiwan (Figure 9A). A case of human infection with the virus from this clade in 2013 prompted research into the structure and receptor-binding properties of HA [for a review, see Everest et al. (2020)]. One of the reports predicted that the substitutions E190V and G228S in the HA RBS, in combination with N137, could allow the H6N1 viruses to bind to human-type receptors (Ni et al., 2015). In this view, we generated a phylogenetic tree of the H6N1 NA annotated with amino acids 137, 190, and 228 of the corresponding HAs (Figure 9B). According to the phylogeny, the Y347F substitution emerged and was fixed in parallel with unique substitutions in HA, R137N and either E190L or E190V + G228S.

In the H5av-1 group, the non-Y NA variants were scattered across different clades of Gs/Gd-like IVs (Figure 10A). Occasional clusters of the Y347H and Y347D mutants containing from 2 to 10 sequences were observed, suggesting limited expansion of these mutants. Variants having Q, F, and S were represented by a single sequence each. Two H5hum mutants (Y347H, Y347G) and one H5mam mutant (Y347H) clustered with avian-derived NAs that had Y347 (see the trees in Supplementary material). We next analyzed the phylogeny of H5 HA sequences that included annotations of NA residue 347 and groups of HA residues, that form the RBS. As an example, the part of the phylogeny shown in Figure 10B demonstrates that the cluster of viruses with D347 in the NA differs from the close Y347-containing IVs by 3 amino acid substitutions in HA, namely S137A, D187N, and K193D. Using this analysis, we found association between NA substitutions and HA substitutions in roughly half of the H5N1 virus clusters and single sequences examined (Figure 10A; Table 4). Remarkably, substitutions at most of these positions have been previously shown to affect antigenic and receptor-binding properties of H5 HA (Paulson and de Vries, 2013; Koel et al., 2014; Suttie et al., 2019).

**Table 4:** Substitutions in H5 HA associated with NA substitutions at position 347.

Virus	347	*N*	Substitutions
A/duck/Guangdong/22/2002	H	1	S133L
A/Cambodia/JP52a/2005	H	1	T192I
A/peregrine falcon/Hong Kong/810/2009	D	6	S137A, D187N, K193D*
A/chicken/East Kalimantan/UT581/2010	F	2	L154I, S159N
A/wild duck/Fujian/1/2011	H	2	Q196K + R227S
A/chicken/Bangladesh/3012/2011^#^	D	1	K193Q
A/chicken/Bangladesh/11RS-1984-30/2011^#^	D	1	S185P, D187N, K193Q
A/chicken/Bangladesh/08C178/2016	D	2	D158N (G+), D187N, K193T
A/chicken/Togo/17RS1021-7/2017	H	2	S221P

N, number of HA sequences in the cluster; G+, substitution creates glycosylation sequon; *, D193 was not found in any other H5 HA sequence analyzed; ^#^, NAs belong to the same cluster of 3 NAs in Figure 10.

#### 3.2.4 Selection pressure analyses

These analyses were performed to determine, whether site 347 evolved under natural selection pressure and whether pressures varied depending on the virus host species and/or phylogenetic lineage.

Using the combined alignment of all sequences (*N* = 17,856) we found that site 347 is evolving under overall (pervasive) purifying selection (FEL dN/dS = 0.20, value of p for non-neutral evolution <10^−20^). However, a more sensitive method (MEME) identified that this site also experienced episodic diversifying selection (MEME dN/dS = 8.7, proportion assigned to dN/dS > 1 = 10%, value of p for episodic positive selection = 0.025). Next, using the combined tree, we estimated dN/dS values for subsets of branches partitioned by groups as described above (Figure 7; Table 3) (Note, that we placed H5N1 sequences isolated from mammals or humans in one group to improve statistical power, given the relatively small sizes of each group). We found that 8 out of 10 studied groups of branches evolved subject to purifying selection (*p* < 0.05, LRT with Holm-Bonferroni correction for multiple testing) (Table 5). Overall, unlabeled branches were also subject to purifying selection. As only three substitutions have been accumulated on the combined H5hum + H5mam lineages, there was insufficient statistical power to discern selection. H5av-1 branches encompassed considerably more variation, with 8 synonymous and 16 non-synonymous substitutions, consistent with neutrality (Table 5). However, there was a significant difference (*p* = 0.05, LR test) between the evolutionary regimes along internal branches (dN/dS = 1.3), which, by necessity, represent some host-to-host transmissions, and terminal branches (dN/dS = 0.4), which often reflect within-host evolution. Thus, while we cannot reject the hypothesis of neutrality on all H5av-1 branches, an observation that dN/dS is significantly higher along internal H5av-1 branches, enriched for evolution between hosts (and host species), is consistent with a functional role of this site. We next ran MEME, which is capable of detecting episodic diversifying selection (EDS) affecting only a proportion of branches. Although we detected EDS for the whole NA alignment, no individual group provided statistical support for EDS, likely due to loss of power and relatively few substitutions compared to those accumulated along the entire tree.

**Table 5:** Evolutionary analysis of site 347.

Virus group	BN	dN/dS (CI)	*p*	Inferred substitutions*
Avian HxN1 (x = 1–4, 7–12)	1,211	0.00 (0.00–0.29)	4×10^−6^	Y(5)
Avian H6N1	643	0.14 (0.00–0.89)	0.03	Y(2), Y-*F*(1)
H5av-1	3,685	0.72 (0.36–1.27)	0.66	Y(8), Y-H(10), Y-D(3), Y-F(1), Y-Q(1), Y-S(1)
H5av-2	2,993	0.21 (0.02–0.86)	0.03	Y(1), Y-H(2)
H5hum + H5mam	618	1.25 (0.24–3.62)	0.68	Y-G(1), Y-H(2)
Classical swine	4,866	0.11 (0.03–0.29)	9×10^−10^	N(8), N-D(5)
ALS-1	2,603	0.02 (0.00–0.10)	4×10^−6^	N(16), N-D(1)
ALS-2	1,787	0.23 (0.06–0.61)	7×10^−4^	N (5), N-D(1), N-K(4)
Seasonal	3,383	0.42 (0.14–0.93)	0.03	N(9), D(2), D-N(5), N-D(3)
H1N1pdm, representative	3,295	0.09 (0.01–0.36)	2×10^−6^	N(5), N-D(1), N-K(1)
Unlabeled	1,570	0.24 (0.05–0.70)	0.035	Y(7), N(1), D-N(1), N-D(1), N-Y(1), Y-N(1)

dN/dS is estimated using FEL, and profile likelihood confidence intervals (CI, 99%) are shown. The value of p for non-neutral evolution is obtained under a likelihood ratio test, where only the dN/dS ratio for this group is constrained to be 1 in the combined tree with all sequences; the Holm-Bonferroni correction for multiple testing is applied. Substitutions are inferred based on the joint maximum likelihood ancestral codon reconstruction. BN, number of branches tested; P, value of p for dN/dS ≠ 1; *, X(n), n synonymous substitutions for amino acid X; X-Z(n), n non-synonymous substitutions from amino acid X to amino acid Z.

We also interrogated the entire NA alignment for evidence of sites that may be co-evolving with site 347 using a Bayesian Graphical Model phylogenetic method (Poon et al., 2007). No evidence of interactions among site 347 and other sites in NA was found.

In summary, negative selection within most groups of viruses indicates that the amino acids at position 347 specifically adapted to particular hosts are maintained, whereas episodic positive selection across the entire tree is consistent with the hypothesis that adaptation occurs during or following some host switches.

## 4 Discussion

The N1 NAs of avian IVs hydrolyze the Neu5Ac2–6Gal linkage much less efficiently than the Neu5Ac2–3Gal linkage. In contrast, N1 NAs of swine and human IVs show less restricted linkage specificity due to increased activity against the 2–6 linkage and diminished activity against the 2–3 linkage (Mochalova et al., 2007; Gerlach et al., 2012). Previous molecular dynamics simulation studies on N1 NAs from phylogenetically distant avian and human IV isolates suggested that amino acid 347 contributed to the specificity of the enzyme for the Neu5Ac-Gal linkage type (Raab and Tvaroska, 2011; Jongkon and Sangma, 2012; Phanich et al., 2019). Here, we explored this hypothesis by modeling the interaction between sialoglycan substrates and two N1 NAs that differed only by amino acid 347.

Comparison of the complexes of avian-type NA/Y with 3S and 6S revealed the following differences. First, 3S bound to NA/Y in one of its solution-populated conformations, while 6S was forced to adopt a novel conformation that was not populated in its unbound state (Figure 2). Second, the Neu5Ac moiety of the bound 3S was located in the center of the NA catalytic pocket, with optimal contact distances of Neu5Ac to the arginine triad and E276 [residues known to make the major contributions to the binding energy (Taylor and von Itzstein, 1994)], and to the key catalytic residues, such as Y406. In contrast, the Neu5Ac moiety of 6S was tilted in the complex, resulting in a network of contacts that differed from those in both 3S-NA/Y and crystal structures of NA complexes with free Neu5Ac (Table 1; Figures 3A,B). Third, the estimated binding free energy was less favorable in the case of 6S (Table 2). Fourth, catalysis-promoting distortion of the Neu5Ac ring from chair to pseudo-boat was observed in the 3S-NA/Y complex, with little, if any, distortion evident in the 6S-NA/Y complex (Figure 5). Finally, the solvation of the glycosidic bond was less efficient in the 6S-NA/Y complex (Figure 6). All of these differences are expected to decrease the binding avidity and hydrolysis efficiency of 6S in comparison to 3S. Remarkably, the Y347N substitution alleviated most of the mentioned adverse effects by allowing the binding of 6S to NA/N in a solution-populated conformation, improving the orientation of the Neu5Ac moiety and the solvation of the glycosidic linkage, and considerably enhancing the free energy of binding. At the same time, replacement of Y with N negatively impacted binding characteristics of 3S. These findings advance and substantiate previous notions about the significance of amino acid 347 for the NA recognition of the Neu5Ac-Gal linkage. However, substrate binding represents only the first step of the NA-mediated catalysis (Taylor and von Itzstein, 1994). Although catalytic activity correlates with binding affinity (Mochalova et al., 2007; Garcia et al., 2013), other steps, such as donation of the proton from the solvent, formation of the endocyclic sialoside cation intermediate, and release of the asialic part of the substrate may also depend on the type of the Neu5Ac-Gal linkage. Therefore, further analyses of the catalytic activity of the 347 mutants are required to refine the predictions made by modeling.

Our MD simulation data suggest the following molecular mechanisms for the effect of residue 347 on substrate specificity of the NA. We found that optimal binding of 3S to NA/Y depends on favorable polar interactions between the 4-OH group of Gal and the OH group of Y347. In this conformation of bound 3S, the 2-hydroxyl of Gal forms an H-bond with the main chain carbonyl group of V149, while GlcNAc makes a van der Waals contact with the side chain of V149 (Figures 1A, 3A; Table 1). In the case of 6S, the Neu5Ac2–6Gal moiety approaches the side chain of Y347 with the apolar side of the Gal residue. To avoid a steric conflict and unfavorable polar-apolar interactions between Gal and the OH group of Y347, the glycan is shifted toward loop 150; this shift negatively affects interactions with key catalytic residues and reduces binding avidity. In contrast to NA/Y, the less bulky side chain of N347 in the NA/N variant allows accommodation of the Neu5Ac2–6Gal moiety in the catalytic pocket in one of its solution-populated conformations. Furthermore, the 6S-NA/N complex is stabilized by polar contacts of the 3-OH and 4-OH groups of Gal with the side chain of N347 and by interactions of GlcNAc and Gal2 with P431 and Q430, respectively (Table 1). The unfavorable effect of the Y347N substitution on NA complex with 3S can be explained by the loss of stabilizing polar interactions between Gal and residue 347. To compensate for this loss, the glycan tilts and shifts toward loop 150, increasing interactions of the Gal and GlcNAc residues with V149 (Table 1).

The initial notion about the association of amino acid 347 with the IV host range (Fanning et al., 2000) was based on the analysis of a small number of N1 NAs and, to the best of our knowledge, has not been followed by systematic analysis. To fill this gap, we assessed the identity of residue 347 in all currently available N1 NA sequences of IVs in different host species. Remarkably, with two exceptions, Y347 was perfectly conserved in avian IVs regardless of their HA subtype, while N347 was highly conserved in classical swine, avian-like swine, and H1N1pdm virus lineages (Table 3). Natural selection analyses based on dN/dS ratios suggested that position 347 was under pervasive purifying selective pressure in these avian and mammalian viruses (Table 5). The NAs of the avian-origin 1918 pandemic virus and its successors isolated in 1933–1947 also contained N347 (Figure 8A). Combined with the modeling data, these results indicate that efficient cleavage of 2-3-linked sialoglycans associated with Y347 is essential for avian IV fitness, and that the Y347N substitution and resulting changes in substrate specificity of NA are essential for avian virus adaptation to humans and pigs. This pattern correlates perfectly with changes in HA receptor-binding specificity during avian-to-human and avian-to-swine transmission (Matrosovich et al., 2006; Shi et al., 2014; Thompson and Paulson, 2020; Liu et al., 2023).

Interestingly, a functional similarity can be observed between Y347 of N1 NA and conserved Q226 of the avian HA. First, both Q226 of HA and Y347 of NA play important roles in protein binding to one of the solution-dominant conformations of Neu5Ac2–3Gal-terminated receptors via interactions with the 4-hydroxyl group of Gal (Supplementary Figure S3). Second, both residues hinder the accommodation of the Gal residue in solution-dominant conformers of Neu5Ac2–6Gal-terminated receptors. Third, the Q226L substitution in avian influenza viruses of diverse HA subtypes occurs during the virus adaptation to humans and swine and alters its preference for the type of Neu5Ac-Gal linkage.

The first viruses of the ALS lineage isolated in 1979 carried substitutions E190D and/or G225E in the HA and bound to both Neu5Ac2-3Gal- and Neu5Ac2-6-Gal-terminated receptors. The binding preference of the virus for 6-linked receptors and transmissibility in pigs under experimental conditions increased over time (Ito et al., 1998; Matrosovich et al., 2000; Su et al., 2021). Because the Y347N substitution appeared and became fixed after 1981 (Figure 8B), we conclude that it was not essential for the initial avian-to-swine transmission and was selected at a later stage, likely, as a compensation for the increased HA avidity for Neu5Ac2–6Gal.

Seasonal human IVs exhibited a distinct pattern of NA evolution with an initial circulation of N347-containing IVs, their replacement by the D347 variants, and the periodic emergence of new N347-containing lineages (Figure 8A). The D347N substitution was previously found to increase NA avidity and catalytic activity with respect to the synthetic substrate MUNANA (Collins et al., 2009; Rameix-Welti et al., 2011; Duan et al., 2014). Collins et al. (2009) observed this effect in IVs from three independent clades of D-to-N mutants. As suggested by the authors, the substitution increased substrate binding by eliminating unfavorable electrostatic interactions between the negatively charged carboxylic groups of Neu5Ac and D347. It has been speculated that the exchange of N and D at position 347 and corresponding changes in NA catalytic activity reflect epistatic interactions of amino acid 347 with other amino acids of NA and/or with the HA (Collins et al., 2009; Duan et al., 2014). For example, either D or N may be selected to compensate alterations of NA catalytic activity and/or HA receptor-binding activity of the antigenic drift variants of these proteins. Although we found no evidence of interactions between residue 347 and other sites in NA, further studies are needed to test this hypothesis and to characterize underlying mechanisms of N/D switching in at the NA position 347 in seasonal IVs.

Most avian IVs bind to Neu5Ac2–3Gal-terminated receptors, however, avian viruses may differ in the ability to recognize the sub-terminal saccharide parts of the sialoglycans. Thus, mallards and other dabbling ducks (*Anatinae*) carry all IV subtypes except H13 and H16 and are thought to play a key role in the maintenance of IVs in nature (Fouchier and Munster, 2009; Verhagen et al., 2021). Duck-adapted IVs exhibit similar receptor-binding traits, in particular, share a preference for binding to Neu5Ac2–3Gal1-3/4GlcNAc-containing receptors, with low tolerance for fucosylation of the GlcNAc moiety [(Gambaryan et al., 2012, 2018) and references therein]. Observed conservation of the NA residue Y347 in avian IVs with HA subtypes H1-H4 and H7-H12 (Tables 3, 5) is consistent with the high conservation of the HA RBS and receptor specificity of IVs in ducks.

Adaptation of aquatic bird viruses to land-based gallinaceous birds, such as chicken and quail, is often accompanied by substitutions in the HA and alteration of the receptor-binding specificity. In contrast to duck IVs, poultry-adapted viruses typically show high-avidity binding to Neu5Acα2–3Galβ1–4GlcNAc-terminated receptors containing fucose and/or sulfate at the GlcNAc residue. Moreover, some of these viruses, for instance, certain H9N2 and H7N9 lineages, acquire substitutions at the conserved positions of the RBS, such as 190, 225, and 226, which facilitate HA binding to Neu5Ac2–6Gal-terminated receptors [for reviews, see Matrosovich et al. (2008), Sriwilaijaroen and Suzuki (2020), Thompson and Paulson (2020), and Zhao and Pu (2022)]. Remarkably, two groups of poultry-adapted IVs studied here differed from the other avian IVs by the presence of substitutions at NA position 347.

IVs with the H6 subtype HA have a distinctive receptor-binding specificity (Gambaryan et al., 2018) and the broadest host range in birds compared to other IV subtypes (Everest et al., 2020). The Y347F substitution emerged on a single occasion in the chicken H6N1 viruses in parallel with the substitutions at HA positions 137, 228 and/or 190 (Figure 9; Table 5). There is no consensus between different research groups about the effect of these substitutions on receptor-binding specificity. Two studies concluded that the substitutions increase HA binding to Neu5Ac2–6Gal-terminated receptors (Ni et al., 2015; Wang et al., 2015), and one study showed that the substitution E190V increased HA binding to sulfated species of Neu5Ac2–3Gal-containing receptors (Kikutani et al., 2020). In any case, the non-conservative substitutions in the canonical conserved positions of the avian RBS should significantly alter receptor specificity of these H6N1 IVs.

Highly pathogenic H5N1 avian IVs with the Gs/Gd-like HA emerged in 1996 in Southern China, spread to other countries and have been causing continuous outbreaks in poultry and wild birds with occasional infections of mammals including humans. These IVs diverged into multiple H5 HA clades with different NA subtypes (Lycett et al., 2019; Demirev et al., 2023; Yang et al., 2023). Due to the wide geographical spread of H5N1 IVs, their broad host range and tissue tropism, as well as immune pressure imposed by vaccination of poultry, a large number of substitutions in the vicinity of the HA RBS were selected that affected both antigenicity and receptor-binding properties of the HA (Paulson and de Vries, 2013; Koel et al., 2014; Suttie et al., 2019). Most of these substitutions likely reflect antigenic drift and/or adaptation of the HA to different species- and tissue-specific Neu5Ac2–3Gal-containing receptors in wild and domestic avian hosts (Kuchipudi et al., 2021; Zhao adn Pu, 2022); some of the substitutions marginally increase HA avidity for human-type receptors. We found that the Gs/Gd-like lineage contained a number of independently emerging NA mutants (typically with H347 and D347), and that the substitutions in the NA were associated with substitutions in or near the HA RBS (Table 4). For example, all three different NA clusters with D347 shown in Figure 10A carried, among other substitutions, the HA substitution at position 193 from K to D, Q or T. A positively charged amino acid (K/R) at this site is responsible for the high-avidity binding of H5, H7, H13 and H16 subtype IVs to sulfated sialoglycans, such as Su-3SLN and Su-SLex (Gambaryan et al., 2012, 2018, and references therein). Therefore, the substitution of K193 with either a negatively charged or uncharged amino acid should have a significant effect on the receptor-binding properties of H5 HA. It is noteworthy that the Y347H NA mutants had different substitutions in the HA compared to the Y347D mutants, indicating the probable functional interplay between the HA and NA substitutions in these H5N1 IVs. We conclude that the substitutions at position 347 in the NA of both H5N1 and H6N1 were selected to alter the NA catalytic activity and restore the HA/NA balance that was disrupted by the substitutions in the HA. However, it cannot be ruled out that the NA substitutions emerged first, followed by selection of corresponding compensatory substitutions in HA.

In summary, our molecular modeling data combined with the analyses of NA and HA sequences strengthen previous observations on the role of amino acid 347 in the N1 NA recognition of the Neu5Ac-Gal linkage, predict the underlying molecular mechanisms, and suggest that substitutions at NA position 347 represent a novel marker of viral host range, interspecies transmission, and adaptive evolution. These findings call for further studies, including in-depth analyses of the catalytic activity and epistatic interactions of position-347 N1 NA mutants as well as identification of analogous host-specific substitutions within the catalytic domain of other NA subtypes.

## Data availability statement

The original contributions presented in the study are included in the article/Supplementary material, further inquiries can be directed to the corresponding authors.

## Author contributions

MM conceived and planned the structure of the article and supervised the whole project. MM and MG acquired funding. SE, GR, and MG generated and analyzed MD simulation data, MM prepared the sequence datasets and performed phylogenetic analyses, SKP performed selection analyses. SE, SKP and MM analyzed the results, prepared the figures and tables and wrote the manuscript. All authors contributed to the article and approved the final version of the manuscript.

## Footnotes

^1^ https://tree.bio.ed.ac.uk
