# Supplementary material for: Molecular modeling and phylogenetic analyses highlight the role of amino acid 347 of the N1 subtype neuraminidase in influenza virus host range and interspecies adaptation
Source: Front Microbiol. 2023 Dec 19;14:1309156. doi: 10.3389/fmicb.2023.1309156 (PMC10758481; doi:10.3389/fmicb.2023.1309156)
Supplement: Supplementary file 1 [file Data_Sheet_1.ZIP › Supplementary materials.docx]

***Supplementary Material***

Molecular modeling and phylogenetic analyses highlight the role of amino acid 347 of the N1 subtype neuraminidase in influenza virus host range and interspecies adaptation

Stefano Elli, Giuseppina Raffaini, Marco Guerrini, Sergei Kosakovsky Pond, Mikhail Matrosovich


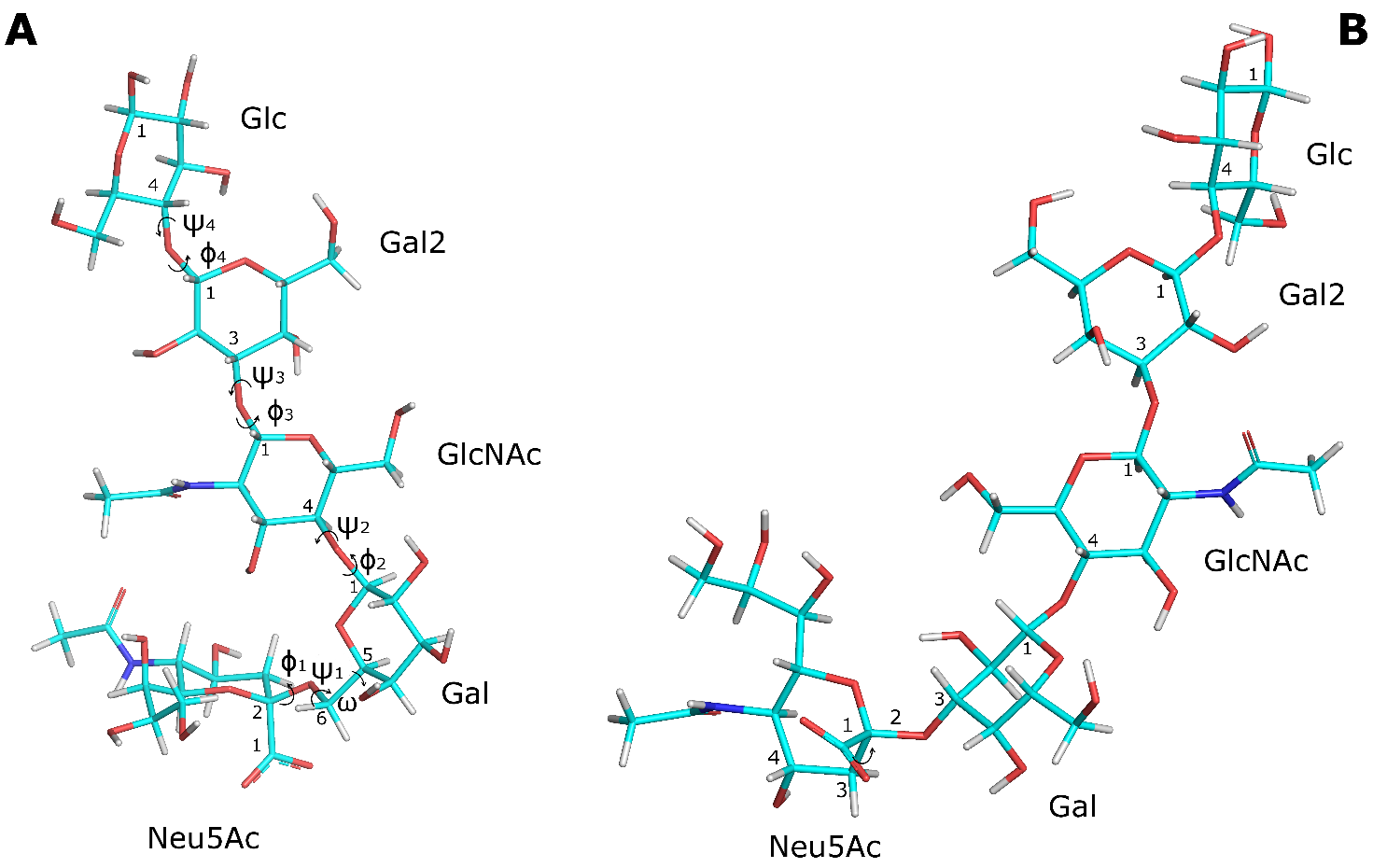


**Supplementary Figure S1**

Stick models of pentasaccharides α-D-Neu5Ac(2→6)β-D-Gal(1→4)β-D-GlcNAc-(1→3)β-D-Gal(1→4)β-D-Glc-OH (6S, **panel A**) and α-D-Neu5Ac(2→3)β-D-Gal(1→4)β-D-GlcNAc(1→3)β-D-Gal(1→4) β-D-Glc-OH (3S, **panel** **B**). Carbon, nitrogen, oxygen and hydrogen atoms are colored in cyan, blue, red and white, respectively. The Neu5Ac2-3Gal linkage of 3S is defined by dihedral angles φ_1_ (C1-C2-O2-C3) and ψ_1_ (C2-O3-C3-H3). The Neu5Ac2-6Gal linkage of 6S is defined by φ_1_ (C1-C2-O6-C6), ψ_1_  (C2-O6-C6-C5) and ω (O6-C6-C5-H5). The other pairs of glycosidic dihedral angles are defined by consecutive atoms H_i_-C_i_-O_i+1_-C_i+1_ and C_i_-O_i+1_-C_i+1_-H_i+1_. The intra-residue dihedral angle C1-C2-C3-C4 determines the chair/boat conformations of Neu5Ac. Indicated designations of monosaccharide residues are used throughout the text.


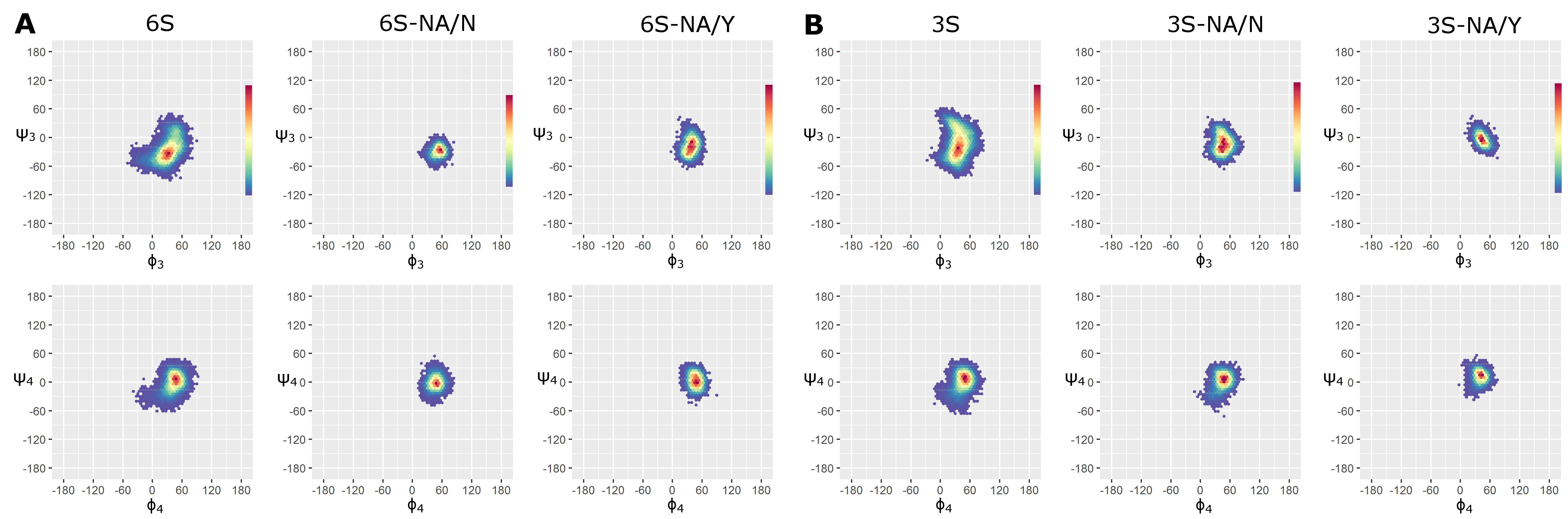


**Supplementary Figure S2**

Ramachandran plots and color density map of the glycosidic linkages φ_3_/ψ_3_ and φ_4_/ψ_4_ of the pentasaccharides 6S (**A**) and 3S (**B**) in their unbound state ((Elli et al., 2021) with permission from the Biochemical Journal) and in complex with the NA/N and NA/Y. The color gradient from blue to red on each map is proportional to the increase in population density of the states sampled by MD simulation.

**
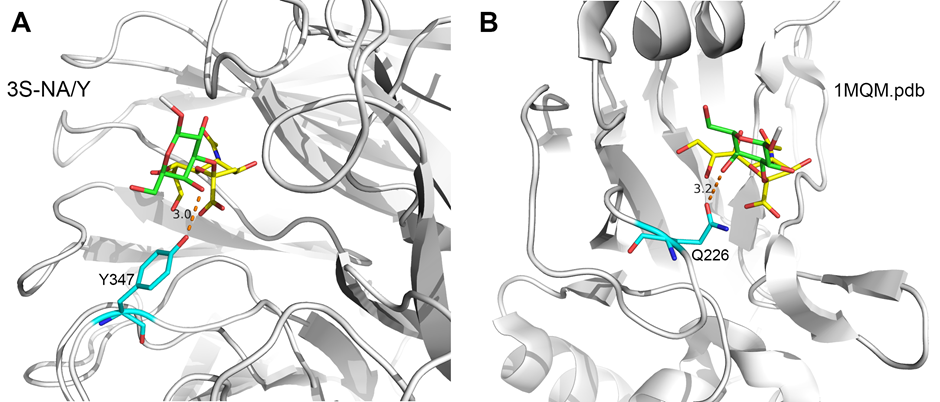
**

**Supplementary Figure S3**

Structure of the 3S-NA/Y complex (**A**) and the crystal structure of the avian H3 HA complex with sialoglycan LSTa (1MQM) (Ha et al., 2003) (**B**). The terminal Neu5Ac2-3Gal moieties of the sialoglycans and amino acids Y347 (NA) and Q226 (HA) are shown as stick models. The carbon atoms of these residues are colored in yellow (Neu5Ac), green (Gal) and cyan (Y347, Q226). Oxygen and nitrogen atoms are colored in red and blue, respectively. Orange dashed lines show bonds between the amino acids and the 4-hydroxyl group of Gal. The structures are aligned based on the coordinates of the Neu5Ac residues.

**Supplementary Table S1**

The most populated glycosidic dihedral angles φ_i_/ψ_i_ and ω estimated from the Ramachandran plots and the density color maps of free 6S and 3S as well as their complexes with NA/N and NA/Y. The percentage of the φ_1_/ψ_1_ states populated by the glycosidic linkage of Neu5Ac-Gal in the unbound state are given in parentheses. The φ_1_/ψ_1_ states populated in both the free and bound states are shown in bold.

| **Dihedral angles** | **6S*** | **6S-NA/N** | **6S-NA/Y** | **3S*** | **3S-NA/N** | **3S-NA/Y** |
| --- | --- | --- | --- | --- | --- | --- |
| φ_1_/ψ_1_ | -62/-163 (41%),  -73/155 (26%),  **-66/110 (20%)**,  -173/170 (13%) | **-67/122** | 52/-130 | -67/-3 (74%),  **-92/-58 (15%)**,  -168/-23 (11%) | -55/-100 | **-79/-42** |
| φ_2_/ψ_2_ | 45/0 | 49/27 | 36/23 | 48/10 | 24/-26 | 36/18 |
| φ_3_/ψ_3_ | 31/-33 | 57/-25 | 38/-20 | 37/-23 | 48/-9 | 43/-2 |
| φ_4_/ψ_4_ | 47/5 | 48/-2 | 48/1 | 49/8 | 48/8 | 42/15 |
| ω | -50 | -3 | 173 | - | - | - |

* Data for free 6S and 3S previously reported in (Elli et al., 2021) are presented here with permission from the Biochemical Journal.

**Supplementary trees. Legends.**

The trees are provided in the nexus and svg formats. ­­

**Files** **Global NA tree.nex** and **Global NA tree.svg**

Phylogenetic relationships of 18097 N1 NA gene sequences from the GISAID EpiFlu database used in this study. For the H1N1pdm lineage, only 2568 representative sequences out of the total 32752 sequences were included in the tree.

The taxa names, such as H5av_1_quail_Vietnam_NCVD_3794_2013_A___H5N1__898179___Y, include the sequence group, virus name, subtype, EpiFlu accession number and a single letter code of amino acid at NA position 347. The maximum likelihood tree was generated using the FastTree method (Price et al., 2010) included in Unipro UGENE 47.0 (Okonechnikov et al., 2012).

**Files** **H5N1 HA tree.nex** and **H5N1 HA tree.svg**

Phylogenetic relationships of 2932 HA sequences of H5N1 viruses isolated prior to January 1, 2020, where the complete NA sequences were also available in the GISAID EpiFlu database. The taxa names, such as A_duck_Jiangxi_120_2011___D--LGVSAA-WLI-NNT-SNNEAEQTDLYQN-SKVNGQSG, include the virus name followed by NA residue 347 and groups of HA residues 133-138, 153-155, 158-160, 185-197, 221-228 separated by minus sign. The maximum likelihood tree was generated using FastTree. The names of viruses containing the 347 NA mutants are colored in red.

**Supplementary references**

Elli, S., Gambacorta, N., Rudd, T.R., Matrosovich, M., and Guerrini, M. (2021). MD simulation of the interaction between sialoglycans and the second sialic acid binding site of influenza A virus N1 neuraminidase. *Biochem J* 478(2)**,** 423-441. doi: 10.1042/BCJ20200670.

Ha, Y., Stevens, D.J., Skehel, J.J., and Wiley, D.C. (2003). X-ray structure of the hemagglutinin of a potential H3 avian progenitor of the 1968 Hong Kong pandemic influenza virus. *Virology* 309(2)**,** 209-218. doi: doi.org/10.1016/S0042-6822(03)00068-0.

Okonechnikov, K., Golosova, O., Fursov, M., and team, U. (2012). Unipro UGENE: a unified bioinformatics toolkit. *Bioinformatics* 28(8)**,** 1166-1167. doi: 10.1093/bioinformatics/bts091.

Price, M.N., Dehal, P.S., and Arkin, A.P. (2010). FastTree 2 – approximately maximum-likelihood trees for large alignments. *PLOS ONE* 5(3)**,** e9490. doi: 10.1371/journal.pone.0009490.
